# 5-Aminolevulinic acid improves cold resistance through regulation of SlMYB4/SlMYB88-SlGSTU43 module to scavenge reactive oxygen species in tomato

**DOI:** 10.1093/hr/uhae026

**Published:** 2024-01-19

**Authors:** Zhengda Zhang, Luqiao Yuan, Jiao Dang, Yuhui Zhang, Yongshuai Wen, Yu Du, Yufei Liang, Ya Wang, Tao Liu, Tianlai Li, Xiaohui Hu

**Affiliations:** College of Horticulture, Northwest A&F University, Yangling, Shaanxi 712100, China; Key Laboratory of Protected Horticulture Engineering in Northwest, Ministry of Agriculture and Rural Affairs, Yangling, Shaanxi 712100, China; Shaanxi Protected Agriculture Research Centre, Yangling, Shaanxi 712100, China; College of Horticulture, Northwest A&F University, Yangling, Shaanxi 712100, China; Key Laboratory of Protected Horticulture Engineering in Northwest, Ministry of Agriculture and Rural Affairs, Yangling, Shaanxi 712100, China; Shaanxi Protected Agriculture Research Centre, Yangling, Shaanxi 712100, China; College of Horticulture, Northwest A&F University, Yangling, Shaanxi 712100, China; Key Laboratory of Protected Horticulture Engineering in Northwest, Ministry of Agriculture and Rural Affairs, Yangling, Shaanxi 712100, China; Shaanxi Protected Agriculture Research Centre, Yangling, Shaanxi 712100, China; College of Horticulture, Northwest A&F University, Yangling, Shaanxi 712100, China; Key Laboratory of Protected Horticulture Engineering in Northwest, Ministry of Agriculture and Rural Affairs, Yangling, Shaanxi 712100, China; Shaanxi Protected Agriculture Research Centre, Yangling, Shaanxi 712100, China; College of Horticulture, Northwest A&F University, Yangling, Shaanxi 712100, China; Key Laboratory of Protected Horticulture Engineering in Northwest, Ministry of Agriculture and Rural Affairs, Yangling, Shaanxi 712100, China; Shaanxi Protected Agriculture Research Centre, Yangling, Shaanxi 712100, China; College of Horticulture, Northwest A&F University, Yangling, Shaanxi 712100, China; College of Horticulture, Northwest A&F University, Yangling, Shaanxi 712100, China; College of Horticulture, Northwest A&F University, Yangling, Shaanxi 712100, China; College of Horticulture, Shenyang Agricultural University, Shenyang 110866, China; College of Horticulture, Shenyang Agricultural University, Shenyang 110866, China; College of Horticulture, Northwest A&F University, Yangling, Shaanxi 712100, China; Key Laboratory of Protected Horticulture Engineering in Northwest, Ministry of Agriculture and Rural Affairs, Yangling, Shaanxi 712100, China; Shaanxi Protected Agriculture Research Centre, Yangling, Shaanxi 712100, China

## Abstract

Cold stress severely affects the growth and quality of tomato. 5-Aminolevulinic acid (ALA) can effectively improve tomato's cold stress tolerance. In this study, a tomato *glutathione S-transferase* gene, *SlGSTU43,* was identified*.* Results showed that ALA strongly induced the expression of *SlGSTU43* under cold stress*. SlGSTU43*-overexpressing lines showed increased resistance to cold stress through an enhanced ability to scavenge reactive oxygen species. On the contrary, *slgstu43* mutant lines were sensitive to cold stress, and ALA did not improve their cold stress tolerance. Thus, *SlGSTU43* is a key gene in the process of ALA improving tomato cold tolerance. Through yeast library screening, SlMYB4 and SlMYB88 were preliminarily identified as transcription factors that bind to the *SlGSTU43* promoter. Electrophoretic mobility shift, yeast one-hybrid, dual luciferase, and chromatin immunoprecipitation assays experiments verified that SlMYB4 and SlMYB88 can bind to the *SlGSTU43* promoter. Further experiments showed that *SlMYB4* and *SlMYB88* are involved in the process of ALA-improving tomato's cold stress tolerance and they positively regulate the expression of *SlGSTU43*. The findings provide new insights into the mechanism by which ALA improves cold stress tolerance. *SlGSTU43*, as a valuable gene, could be added to the cold-responsive gene repository. Subsequently, it could be used in genetic engineering to enhance the cold tolerance of tomato.

## Introduction

Global climate change has become unpredictable in recent years because of the influence of human activities [3], and the frequency of extreme low-temperature events has increased. Cold stress is the primary abiotic stress that hinders plant growth and has detrimental effects on crop yield and quality [17]. Tomato (*Solanum lycopersicum* L.), as a cold-sensitive cash plant [54], is widely cultivated around the world and has an important place in international trade [42].

In investigations into plant cold resistance, researchers have discovered that the application of exogenous regulatory substances, such as 5-aminolevulinic acid (ALA; [10]), melatonin [50], abscisic acid [31], salicylic acid [44], exogenous silicon [21], and jasmonic acid [2], can significantly alleviate the effects of abiotic stress on plants. ALA, acting as a growth regulator ubiquitously present in both animals and plants, has been extensively utilized in plant cold resistance processes for its efficient, non-toxic, and easily degradable attributes [47]. Spraying ALA can enhance plant cold tolerance by promoting chlorophyll synthesis [55], improving nutrient uptake capacity [72], regulating hormone levels [49], enhancing reactive oxygen species (ROS) scavenging capacity [73], inducing osmotic regulatory substance synthesis [63], and activating related signal transduction pathways [33]. Most current research related to ALA primarily focuses on the terminal of the regulatory network. The specific molecular mechanisms underlying ALA's role in early plant stress resistance remain unclear, necessitating further investigation by researchers.

Plants have continuously evolved during the process of coping with environmental stress and developed complex and intricate regulatory strategies to rapidly perceive and respond to cold stress [82]. Maintaining the dynamic balance of oxidation–reduction and timely scavenging of excess ROS is important mechanisms for plants to resist cold stress [81]. As an important part of glutathione metabolism [67] and a multifunctional protease encoded by multiple genes, glutathione-*S*-transferase (GST, EC 2.5.1.18) plays a crucial role in plants in ROS clearance [57], detoxification [36], and substance transport [5, 79]. In accordance with gene structure characteristics and protein homology, the *GST* genes in plants are typically divided into 14 subfamilies [56], with tau (U type) and theta (T type) being the most abundant subfamilies. The use of virus-induced gene silencing (VIGS) to silence *GST* in peach can reduce the accumulation of anthocyanins in peach fruit [37]. *GST* overexpression in plants can increase antioxidant contents, and it has been shown to enhance resistance to powdery mildew in *Arabidopsis* [57] and the cold resistance of oilseed rape [38]. Through *GST*-mediated nontarget metabolic resistance, palmer amaranth (*Amaranthus palmeri*) has the ability to detoxify and improves the resistance of palmer amaranth to herbicides [40]. *GST* expression is usually regulated by upstream transcription factors (TFs). The upregulated *TaGST1* expression regulated by WRKY74 enhances the tolerance of wheat to copper stress [27]. *MdGSTF6* promotes the accumulation of anthocyanins, and it is regulated by MdMYB1 in apple [20]. In *Poncirus trifoliata* (L.) Raf., ERF9 was shown to improve trifoliate orange's cold stress tolerance through the regulation of *PtrGSTU17* expression [70]. However, the upstream regulatory mechanism of *SlGSTs* during tomato cold stress remains unclear and needs to be explored. Furthermore, whether *SlGSTs* play a role in regulating the cold tolerance of tomatoes through ALA remains to be further investigated.

In our previous studies, we conducted preliminary investigations into the role of ALA in enhancing tomato cold tolerance. This was carried out using multiple approaches including physiological index and gene expression measurements [72], multi-omics analysis [73], histological staining [34], and gene silencing techniques [33]. In this study, it was found that ALA could induce the expression of *SlGSTs* in tomatoes under cold stress. Specifically, *SlGSTU43* (Solyc09g011630) enhances the cold resistance of tomatoes through the action of ALA. *SlGSTU43* overexpression enhanced the ROS-scavenging ability of tomato seedlings, which was beneficial for improving tomato's cold stress tolerance. After *SlGSTU43* was knocked out in tomato, the sensitivity of tomato to cold stress increased, and ALA did not improve the cold stress tolerance of the *slgstu43* mutant lines. The data showed that *SlMYB4* and *SlMYB88* are involved in the process of ALA's improvement of the tomato cold stress tolerance. Moreover, SlMYB4 and SlMYB88 upregulate *SlGSTU43* expression through binding to the promoter of *SlGSTU43*. Together, the data revealed the mechanism underlying ALA-improved cold stress tolerance of tomato and indicated the application potential of *SlGSTU43* in molecular breeding of tomato for cold stress resistance.

## Results

### ALA improves tomato cold stress tolerance

**Figure 1:**
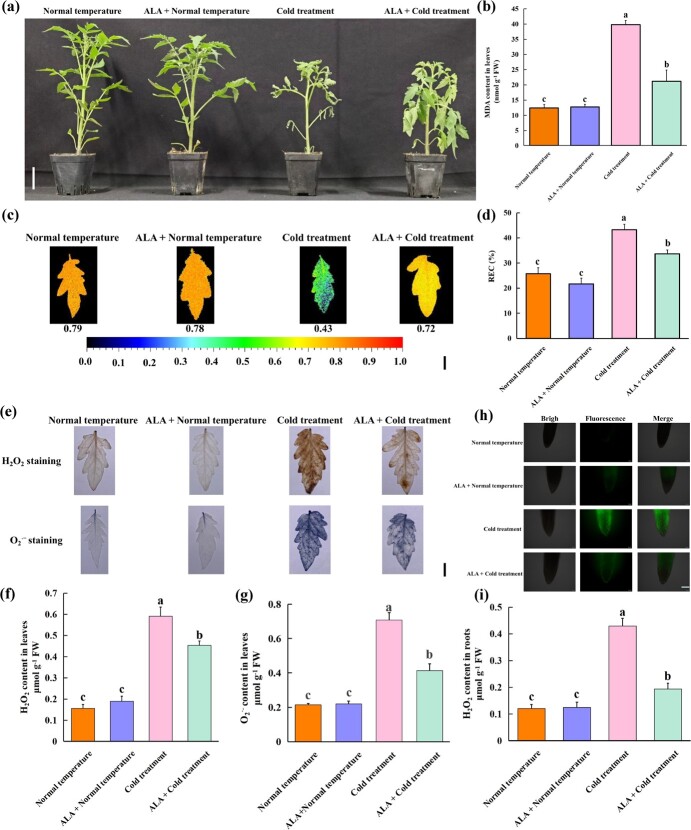
ALA improves tomato cold stress tolerance. **(a)** Phenotype (Scale bar, 5 cm) of tomato. **(b-g)** MDA content (b), Fv/Fm ratio (Scale bars, 1 cm; c), REC ratio (d), histochemical staining (Scale bars, 1 cm; e), H_2_O_2_ content (f), and O_2_^.-^ content (g) in tomato leaves. **(h-i)** H_2_O_2_ accumulation (Scale bar, 100 μm; h) and H_2_O_2_ content (i) in tomato roots. All the indexes and phenotypes of tomato seedlings were obtained after exposure to normal temperature (25°C day/18°C night, 12 h/12 h) and cold stress (4°C day/4°C night, 12 h/12 h) with or without 25 mg·L^−1^ ALA for 8 days. H_2_O_2_ staining with DAB. O_2_^.-^ staining with NBT. H_2_O_2_ accumulation observed with a DCFH-DA probe. The error bars represent ± SDs (*n* = 3). The different letters indicate significant differences (*P* < 0.05) according to Tukey’s test.

After 8 days of cold stress, tomato seedlings became wilted. However, ALA remarkably improved the growth status of tomato seedlings under cold stress (Fig. 1a). Malondialdehyde (MDA) content, the maximum photosystem II efficiency (Fv/Fm) ratio, and the relative electrical conductivity (REC) ratio are commonly used to assess the extent of damage in plants [54, 72]. After cold stress, the MDA content (Fig. 1b) and REC ratio (Fig. 1d) in the leaves of tomato seedlings increased markedly, while the Fv/Fm ratio (Fig. 1c) decreased markedly. ALA significantly increased Fv/Fm ratio and reduced REC ratio and MDA content in leaves of tomato seedlings after cold stress. Combining plant phenotype information and physiological measurements, we confirmed that ALA could alleviate the damage to tomato seedlings caused by cold stress. Cold stress can significantly increase the levels of ROS in plants, thereby causing damage to plants [77]. The contents of H_2_O_2_ (Fig. 1f) and O_2_^.-^ (Fig. 1g) in the leaves and H_2_O_2_ (Fig. 1i) in the roots of tomato seedlings significantly increased after cold stress. In contrast, ALA significantly reduced the contents of H_2_O_2_ and O_2_^.-^ in the leaves of tomato seedlings and the contents of H_2_O_2_ in the roots under cold stress. In addition, we used DAB, NBT, and DCFH-DA probes for histochemical staining of H_2_O_2_ and O_2_^.-^ (Fig. 1e) in leaves and H_2_O_2_ (Fig. 1h) in roots of tomato seedlings. The results of histochemical staining were consistent with those of the ROS content measurements, which also showed that ALA could reduce the accumulation in leaves and roots of tomato seedling under cold stress. Together, these results imply that ALA could enhance the ROS-scavenging ability of tomato seedlings through the activation of a mechanism active during cold stress, thus improving tomato cold stress tolerance.

### ALA induces *SlGSTU43* expression under cold stress

**Figure 2:**
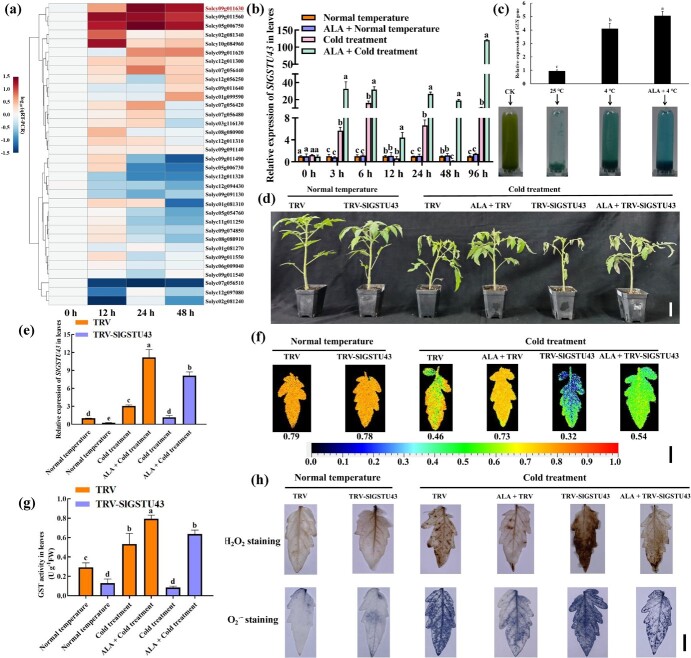
*SlGSTU43 is* involved in the process by which ALA improves tomato cold stress tolerance. **(a)** Expression profiles of 34 *SlGSTs* induced by ALA in tomato seedling leaves under cold stress (4°C day/4°C night, 12 h/12 h) for 0, 12, 24, and 48 h. The red font indicates the target gene (*SlGSTU43,* Solyc09g011630). The concentration of ALA used was 25 mg/L. **(b)***SlGSTU43* expression in tomato seedling leaves after exposure to normal temperature (25°C day/18°C night, 12 h/12 h) and cold stress (4°C day/4°C night, 12 h/12 h) for different durations (0, 3, 6, 12, 24, 48, 96 h) with or without 25 mg/L ALA. **(c)** GUS staining and relative GUS intensity analysis of tobacco (*Nicotiana benthamiana*) under different treatments. A 1747-bp promoter fragment upstream of the start codon (ATG) of *SlGSTU43* was amplified and inserted within the GUS gene in a pBI121 vector to generate pBI121-*SlGSTU43*. CK means tobacco leaves without GUS staining used as a negative control. **(d)** Phenotypes (Scale bar, 5 cm) of TRV and TRV-*SlGSTU43* lines. **(e-h)***SlGSTU43* expression (e), Fv/Fm ratio (Scale bars, 1 cm; f), GST activity (g), and histochemical staining (Scale bars, 1 cm; h) in leaves of TRV and TRV-*SlGSTU43* lines. All the indexes and phenotypes of the tomato seedlings were obtained after exposure to normal temperature (25°C day/18°C night, 12 h/12 h) and cold stress (4°C day/4°C night, 12 h/12 h) with or without 25 mg/L ALA for 6 days. H_2_O_2_ staining with DAB. O_2_^.-^ staining with NBT. The error bars represent ± SD (*n* = 3). The different letters indicate significant differences (*P* < 0.05) according to Tukey’s test.

We validated our previous RNA-seq data [73] using qRT-PCR and found that ALA induced the expression of *SlGSTs* under cold stress. Overall, ALA significantly increased the expression of *SlGSTU43* (Solyc09g011630) in the leaves (Fig. 2a) and roots (Fig. S2) of tomato seedlings under cold stress. To determine the *SlGSTU43* expression pattern, we further tested *SlGSTU43* expression at multiple time points after the start of different treatments. Compared with the normal-temperature treatment, cold stress significantly increased *SlGSTU43* expression at most of the time points that we tested, and ALA further increased *SlGSTU43* expression (Fig. 2b). However, ALA did not alter *SlGSTU43* expression in tomato seedling leaves under normal temperature. In addition, the results of transient expression analysis of the β-glucuronidase (GUS) gene showed that under cold stress, the expression level of *GUS* induced by ALA was the highest (Fig. 2c). This finding further supports the notion that ALA can induce the expression of SlGSTU43 under cold stress. Overall, ALA significantly increased *SlGSTU43* expression in tomato seedlings under cold stress.

We used VIGS technology to silence the *SlGSTU43* gene in tomato plants, aiming to investigate the role of *SlGSTU43* in ALA-mediated improvement of tomato's cold stress tolerance. The mRNA abundance of *SlGSTU43* was drastically reduced in the VIGS-transformed lines (Fig. S1b). After 6 days of cold stress, the TRV-*SlGSTU43* lines were most severely damaged, while ALA improved the phenotype of TRV-*SlGSTU43* lines under cold stress (Fig. 2d). After cold stress, the Fv/Fm ratios (Fig. 2f) in the TRV-*SlGSTU43* lines were the lowest, and the MDA content (Fig. S3a) was the highest, which also indicated that the TRV-*SlGSTU43* lines were more damaged. ALA increased Fv/Fm ratios and decreased MDA content in the TRV-*SlGSTU43* lines under cold stress, which was consistent with the phenotypic results. Under cold stress, the expression of *SlGSTU43* in the TRV-*SlGSTU43* lines was significantly lower than that in the TRV lines (Fig. 2e). ALA significantly increased *SlGSTU43* expression under cold stress, and consistent trends were observed in the TRV and TRV-*SlGSTU43* lines. As *SlGSTU43* expression increased, ALA caused an increase in GST activity in the TRV and TRV-*SlGSTU43* lines (Fig. 2g). As expected, ALA enhanced the ROS-scavenging capacity in the TRV and TRV-*SlGSTU43* lines under cold stress due to the increase in GST activity. According to the results of histochemical staining, cold stress increased the enrichment of H_2_O_2_ and O_2_^.-^ (Fig. 2h) in the leaves of the TRV and TRV-*SlGSTU43* lines and H_2_O_2_ enrichment in the roots (Fig. S3d). ALA could reduce the accumulation of H_2_O_2_ and O_2_^.-^ in the leaves and H_2_O_2_ in the roots of TRV and TRV-*SlGSTU43* lines under cold stress. Similarly, ALA reduced the H_2_O_2_ (Fig. S3b) and O_2_^.-^ contents (Fig. S3c) in the leaves of the TRV and TRV-*SlGSTU43* lines under cold stress and the H_2_O_2_ content in the roots (Fig. S3e). In addition, we noticed that the ROS content of TRV-*SlGSTU43* lines was the highest under cold stress. In summary, our VIGS experiments demonstrated that ALA could reduce the sensitivity of tomato seedlings to cold stress by upregulating the expression of *SlGSTU43* under cold stress. This preliminarily confirmed that *SlGSTU43* was involved in the process through which ALA improves tomato cold stress tolerance.

### Characteristic analysis of *SlGSTU43*

The phylogenetic tree we constructed demonstrates that the U subfamily has the highest number of members among the eight GST subfamilies in tomato (Fig. S4a). Compared with those in rice and *Arabidopsis*, tomato has two unique subfamilies: the MGST subfamily and the Lambda subfamily. We used the SlGSTU43 protein sequence and 49 GST protein sequences of dicotyledons and 31 GST protein sequences of monocotyledons to construct a phylogenetic tree. The analysis results showed that the similarity of the SlGSTU43 protein with GST proteins from willow (MK300944.1) and walnut (KT351091.1) was the highest (Fig. S4b). Meanwhile, we analysed the expression levels of *SlGSTU43* in various tissues of tomato. The results showed that *SlGSTU4*3 had the highest expression in the roots and the lowest expression in the seeds (Fig. S4c). Moreover, conserved motif analysis showed that the SlGSTU43 protein contained three conserved motifs (Fig. S4d). The SlGSTU43 protein tertiary structure was modeled (Fig. S4e), and the resulting Ramachandran plot showed that the model had good structural quality (Fig. S4f).

**Figure 3:**
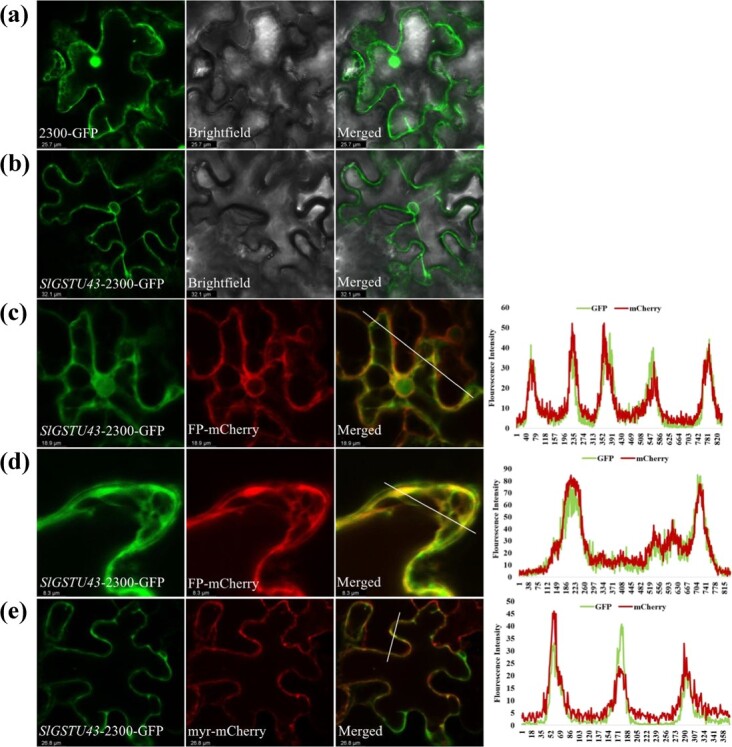
SlGSTU43 is localized in the endoplasmic reticulum and plasma membrane. **(a)** Empty vector (pCAMBIA2300-GFP) transformed into tobacco leaves. **(b)** Fusion construct (pCAMBIA2300-*SlGSTU43*) transformed into tobacco leaves. **(c, d)** Fusion construct (pCAMBIA2300-*SlGSTU43*) cotransformed with an endoplasmic reticulum marker (FP-mCherry) in tobacco leaves. **(e)** Fusion construct (pCAMBIA2300-*SlGSTU43*) cotransformed with a plasma membrane marker (myr-mCherry) in tobacco leaves. The confocal microscopy images of the epidermal cells were taken under brightfield and green (for GFP) and red (for mCherry) fluorescence signals. The fitting of the fluorescence signal was analysed by ImageJ software. The overlapping images are shown on the right. Scale bars = 25 μm.

To confirm the subcellular localization of the SlGSTU43 protein, we coexpressed SlGSTU43 with the endoplasmic reticulum and plasma membrane localization markers FP-mCherry [39] and myr-mCherry [8], respectively. Based on the validated protein markers, the SlGSTU43 protein displayed signals that overlapped with those of FP-mCherry on the endoplasmic reticulum (Fig. 3c, d) and with those of myr-mCherry on the plasma membrane (Fig. 3e). Therefore, SlGSTU43 is localized to the endoplasmic reticulum and plasma membrane.

### *SlGSTU43* positively regulates tomato cold stress tolerance

**Figure 4:**
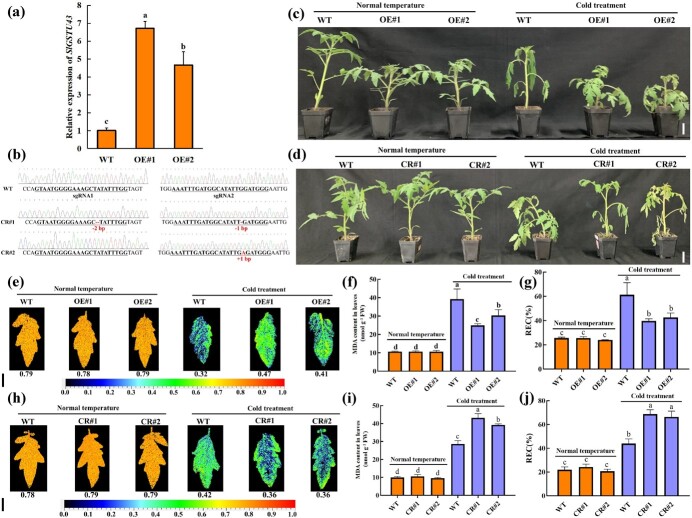
*SlGSTU43* positively regulates tomato cold stress tolerance. **(a)** Transcript levels of *SlGSTU43* in tomato WT and *SlGSTU43*-overexpressing (*SlGSTU43-*OE#1, OE#2) lines. The samples are from the 3rd leaf of *SlGSTU43*-overexpressing transgenic tomato plants at the 3-leaf stage. **(b)** Mutation type of *slgstu43* mutant (*SlGSTU43-*CR#1, CR#2) lines after CRISPR/Cas9-mediated gene editing. There are 2-bp deletions in sgRNA1 and 1-bp deletions in sgRNA2 in CR#1. There is an insertion of 1 A base in sgRNA2 in CR#2. The dashed lines represent nucleotide deletions. **(c, e-g)** Phenotype (Scale bar, 5 cm; c), Fv/Fm ratio (Scale bar, 1 cm; e), MDA content (f), and REC ratio (g) in WT and *SlGSTU43*-overexpressing lines. All the indexes of the tomato seedlings were obtained after exposure to normal temperature (25°C day/18°C night, 12 h/12 h) and cold stress (4°C day/4°C night, 12 h/12 h) for 5 days. **(d, h-j)** Phenotype (Scale bar, 5 cm; d), Fv/Fm ratio (Scale bar, 1 cm; h), MDA content (i), and REC ratio (j) of WT and *slgstu43* mutant lines. All the indexes and phenotypes of the tomato seedlings were obtained after exposure to normal temperature and cold stress for 3 days. The error bars represent ± SD (*n* = 3). The different letters indicate significant differences (*P* < 0.05) according to Tukey’s test.

In order to analyse the function of *SlGSTU43* under cold stress and further investigate whether ALA can improve tomato's tolerance to cold stress through *SlGSTU43*, we generated *slgstu43* mutant lines (CR#1, CR#2) and SlGSTU43-overexpressing lines (OE#1, OE#2). The mRNA levels of *SlGSTU43* in the SlGSTU43-overexpressing lines were significantly higher than those in the wild-type (WT) lines (Fig. 4a). Notably, compared with the WT lines, the *SlGSTU43*-overexpression lines showed obvious dwarfing under normal growth conditions (Fig. S5). The CR#1 mutant had a 2-bp deletion in sgRNA1, and a 1-bp deletion in sgRNA2. In the CR#2 mutant, an A base was inserted into sgRNA2 (Fig. 4b). After 5 days of cold treatment, compared with the overexpression lines, the WT lines exhibited a severely water-soaked-like phenotype (Fig. 4c). Our data showed that the MDA content (Fig. 4f) and REC ratios (Fig. 4g) of *SlGSTU43*-overexpressing lines were significantly lower than those of the WT lines under cold stress, while the Fv/Fm ratios (Fig. 4e) of the *SlGSTU43*-overexpressing lines were significantly higher than those of the WT lines. On the contrary, after cold stress, the wilting phenomenon in *slgstu43* mutant lines was more severe than that in the WT lines (Fig. 4d). The high MDA content (Fig. 4i) and REC ratios (Fig. 4g) and the low Fv/Fm ratios (Fig. 4h) under cold stress also showed that the *slgstu43* mutant lines were subjected to more severe stress than the WT lines were. These results indicate that *SlGSTU43* plays a positive role in improving tomato's tolerance to cold stress. Additionally, they suggest that ALA could improve tomato's tolerance to cold stress by upregulating the expression of *SlGSTU43* under such conditions.

### *SlGSTU43* plays a key role in ALA improving tomato cold stress tolerance

**Figure 5:**
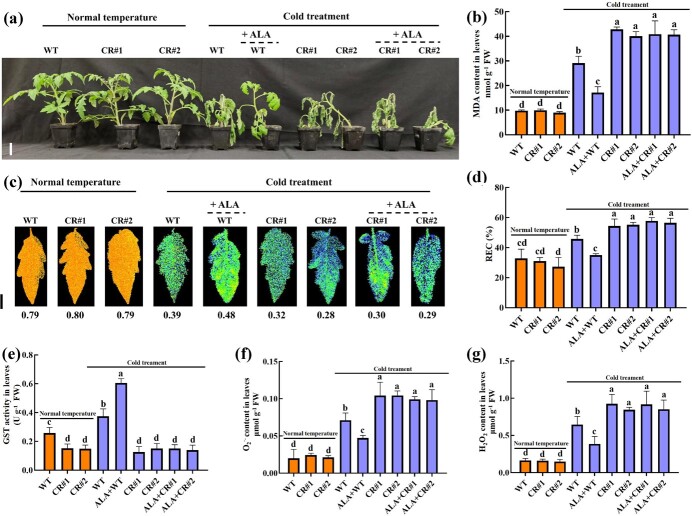
*SlGSTU43* plays a key role in ALA improving tomato cold stress tolerance. **(a-g)** Phenotypes (Scale bar, 5 cm; a), MDA content (b), Fv/Fm ratio (Scale bar, 1 cm; c), REC ratio (d), GST activity (e), H_2_O_2_ content (f), and O_2_^.-^ content (g) in WT and *slgstu43* mutant lines. All the indexes and phenotypes of the tomato seedlings were obtained after exposure to normal temperature and cold stress for 5 days. The error bars represent ± SD (*n* = 3). The different letters indicate significant differences (*P* < 0.05) according to Tukey’s test.

In this study, ALA appeared to improve tomato cold stress tolerance by regulating *SlGSTU43* expression (Fig. 2). We investigated the mechanism of action of ALA by observing the phenotypes of WT and *slgstu43* mutant lines treated with ALA under cold stress (Fig. 5a). The determination of MDA content (Fig. 5b), Fv/Fm ratios (Fig. 5c), and REC ratios (Fig. 5d) also indicates that ALA could not effectively alleviate the damage of *slgstu43* mutant lines under cold stress. These findings suggest that ALA may improve tomato cold stress tolerance through the regulation of *SlGSTU43* expression under cold stress.

In addition, we also measured the GST activity and ROS content of WT and *slgstu43* mutant lines treated with ALA under cold stress. Our results showed that the GST activity of the ALA-treated WT line was higher than that of the WT line under cold stress (Fig. 5e). However, ALA did not alter the GST activity in the *slgstu43* mutant lines under cold stress. With the increase of GST activity, ALA decreased the content of O_2_^.-^ (Fig. 5f) and H_2_O_2_ (Fig. 5g) in the WT line under cold stress. We also noticed that ALA did not reduce the ROS content in the *slgstu43* mutant lines under cold stress. All of our data confirm that *SlGSTU43* is crucial for ALA to improve tomato cold stress tolerance. Under cold stress, ALA could regulate the expression of *SlGSTU43* and increase the GST activity, ultimately improving the cold stress tolerance of tomato seedlings by enhancing their ability to scavenge ROS.

### SlMYB4 and SlMYB88 bind to the *SlGSTU43* promoter and activate its expression

**Figure 6:**
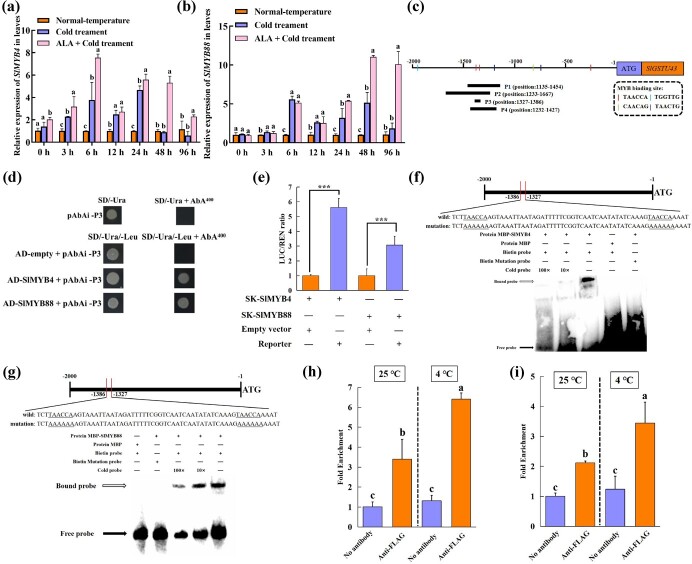
SlMYB4 and SlMYB88 bind to the *SlGSTU43* promoter and activate its expression. **(a-b)***SlMYB4* expression (a) and *SlMYB4* expression (b) in tomato seedling leaves after exposure to normal temperature (25°C day/18°C night, 12 h/12 h) and cold stress (4°C day/4°C night, 12 h/12 h) ALA for different durations (0, 3, 6, 12, 24, 48, 96 h) with or without 25 mg·L^−1^. **(c)** Schematic diagram of *SlGSTU43* promoter. P1 fragment for Y1H assays. P2 fragment for LUC assays. P3 fragment for MESA assays. P4 fragment for ChIP-qPCR assays. **(d)** Y1H assays showing that SlMYB4 and SlMYB88 bind to the promoter region of the *SlGSTU43* promoter. Positive protein-DNA interactions were determined on SD media lacking Ura and Leu but including AbA. **(e)** Various combinations of vectors used in the LUC assays. **(f, g)** EMSA assays showing that SlMYB4 (f) and SlMYB88 (g) proteins directly bind to the biotin-labeled probe of the *SlGSTU43* promoter fragment. **(h, i)** ChIP-qPCR assays revealing the enrichment of SlMYB4 (h) and SlMYB88 (i) in the promoter of *SlGSTU43* via specific primers. The error bars represent ± SD (*n* = 3). The asterisks indicate that the values are significantly different from that of the control (^***^, *P* < 0.001).

We used the *SlGSTU43* promoter fragment as bait to screen prey TFs in a yeast library. According to the annotation results of the tomato genome database, a total of 12 prey TFs were screened (Supplementary Table S7). We then examined the expression patterns of these TFs during the process of enhancing cold tolerance in tomatoes treated with ALA by measuring expression levels. The results showed that ALA significantly increased *SlMYB4* (Solyc09g090130) (Fig. 6a) and *SlMYB8*8 (Solyc05g007160) (Fig. 6b) expression in tomato seedlings under cold stress. Consequently, we chose SlMYB4 and SlMYB88 as candidate prey TFs for further verification.

Yeast one-hybrid (Y1H) assay was carried out to examine whether SlMYB4 and SlMYB88 could activate the *SlGSTU43* promoter. The yeast cells, which were transformed with the prey (SlMYB4 and SlMYB88) and the bait containing P1 fragment, grew well on the medium containing 400 ng/mL AbA, whereas the control group could not grow normally (Fig. 6d), indicating that SlMYB4 and SlMYB88 could bind to the *SlGSTU43* promoter. Similarly, a dual luciferase (LUC) assay was used to further verify the regulatory mechanism of the involvement of SlMYB4 and SlMYB88 with respect to *SlGSTU43*. When the effectors (SK-SlMYB4 and SK-SlMYB88) and the reporter were transiently coexpressed in tobacco leaves, the LUC/REN ratio was significantly higher than that of the control groups (Fig. 6e). We performed an electrophoretic mobility shift (EMSA) to detect whether SlMYB4 and SlMYB88 could bind to the *SlGSTU43* promoter fragment. Incubation with fusion proteins and biotin-labeled probe led to a shift in the protein-DNA complexes (Fig. 6f, g). This phenomenon significantly weakened after the addition of the cold probe. However, we did not detect any binding shift when the biotin-labeled probe was incubated with the MBP protein or when the biotin-labeled mutated probe was incubated with the fusion proteins. Furthermore, chromatin immunoprecipitation (ChIP) assays with FLAG antibody was performed to examine whether SlMYB4 and SlMYB88 could bind to the *SlGSTU43* promoter fragment in tomato. The ChIP-qPCR results showed that SlMYB4 and SlMYB88 were enriched in the P4 fragment of the *SlGSTU43* promoter under normal temperature, and this enrichment was significantly enhanced after cold treatment (Fig. 6h, i). The analysis of *SlGSTU43* promoter cis-acting elements and the selected promoter fragments during subsequent validation process were also presented here (Fig. 6c). Collectively, these results verified that SlMYB4 and SlMYB88 bind to the *SlGSTU43* promoter fragment.

### *SlMYB4* and *SlMYB88* are involved in the process by which ALA improves tomato cold stress tolerance

**Figure 7:**
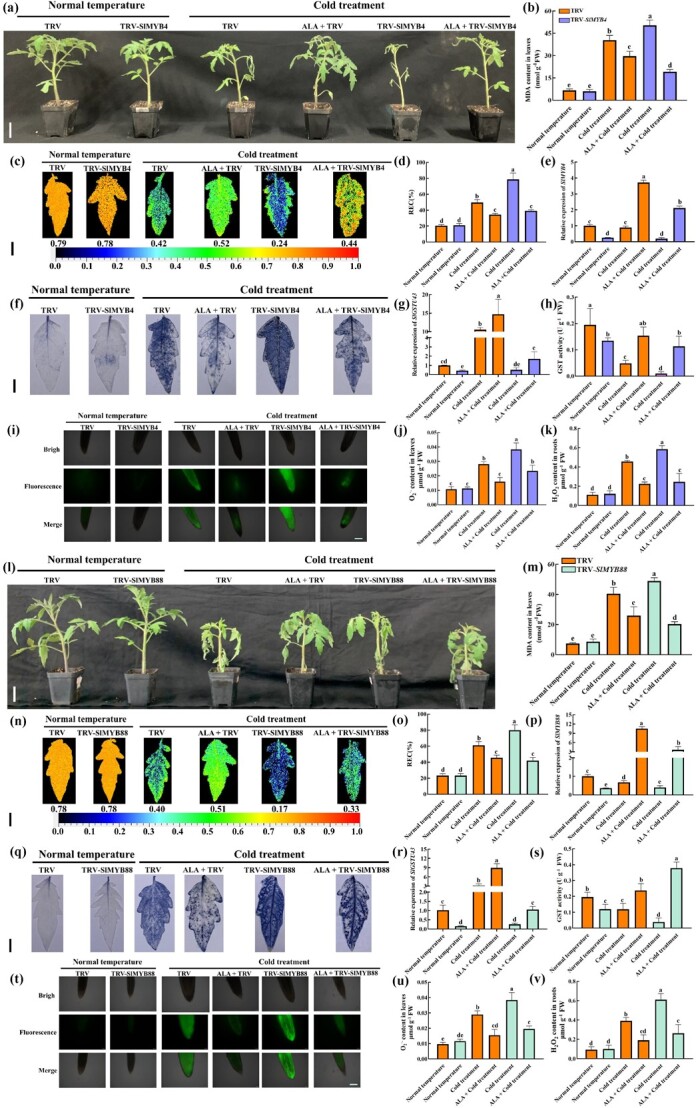
*SlMYB4* and *SlMYB88* are involved in the process by which ALA improves tomato cold stress tolerance. **(a)** Phenotypes (Scale bar, 5 cm) of TRV and TRV-*SlMYB4* lines. **(b-h, j)** MDA content (b), Fv/Fm ratio (Scale bar, 1 cm; c), REC ratio (d), *SlMYB4* expression (e), histochemical staining (Scale bar, 1 cm; f), *SlGSTU43* expression (g), GST activity (h), and O_2_^.-^ content (j) in the leaves of TRV and TRV-*SlMYB4* lines. **(i, k)** H_2_O_2_ accumulation (Scale bar, 100 μm; i) and H_2_O_2_ content (k) in the roots of TRV and TRV-*SlMYB4* lines. **(l)** Phenotypes (Scale bar, 5 cm) of TRV and TRV-*SlMYB88* lines. **(m-s, u)** MDA content (m), Fv/Fm ratio (Scale bar, 1 cm; n), REC ratio (o), *SlMYB88* expression (p), histochemical staining (Scale bar, 1 cm; q), *SlGSTU43* expression (r), GST activity (s), and O_2_^.-^ content (u) in the leaves of TRV and TRV-*SlMYB88* lines. **(t, v)** H_2_O_2_ accumulation (Scale bar, 100 μm; t) and H_2_O_2_ content (v) in the roots of TRV and TRV-*SlMYB88* lines. All measurements for the tomato seedlings were obtained after exposure to normal temperature (25°C day/18°C night, 12 h/12 h) and cold stress (4°C day/4°C night, 12 h/12 h) with or without 25 mg/L ALA for 6 days. O_2_^.-^ staining with NBT. H_2_O_2_ accumulation observed with a DCFH-DA probe. The error bars represent ± SD (*n* = 3). The different letters indicate significant differences (*P* < 0.05) according to Tukey’s test.

To confirm that *SlMYB4* and *SlMYB88* are involved in the process by which ALA improves tomato cold stress tolerance, we silenced *SlMYB4* and *SlMYB88* by the VIGS method. The mRNA abundance of *SlMYB4* and *SlMYB88* was drastically reduced in the VIGS-transformed lines (Fig. S6). Under cold stress, the TRV-*SlMYB4* (Fig. 7a) and TRV-*SlMYB88* (Fig. 7l) lines were remarkably more damaged than the TRV plants were. At the same time, compared with the TRV lines, the TRV-*SlMYB4* and TRV-*SlMYB88* lines presented significant increases in the MDA content (Fig. 7b, m) and the REC ratio (Fig. 7d, o) but a decrease in the Fv/Fm ratio (Fig. 7c, n) under cold stress, which also showed that TRV-*SlMYB4* and TRV-*SlMYB88* lines were more sensitive to cold stress. Under cold stress, spraying ALA rescued the phenotype, decreased the MDA content and REC ratio and increased the Fv/Fm ratio of the TRV-*SlMYB4* and TRV-*SlMYB88* lines. These effects were consistent with the way ALA improved cold tolerance in the TRV-*SlGSTU43* lines.

In addition, ALA increased *SlMYB4* expression in the TRV-*SlMYB4* lines (Fig. 7e) and *SlMYB88* expression in the TRV-*SlMYB88* lines (Fig. 7p) under cold stress. ALA also increased the expression of *SlGSTU43* in the TRV-*SlMYB4* (Fig. 7g) and TRV-*SlMYB88* (Fig. 7r) lines under cold stress. Under the different treatments, the *SlGSTU43* expression pattern in the TRV-*SlMYB4* and TRV- *SlMYB88* lines was similar to that of *SlMYB4* in the TRV-*SlMYB4* lines and that of *SlMYB88* in the TRV- *SlMYB88* lines. This finding supports that *SlMYB4* and *SlMYB88* could regulate *SlGSTU43* expression. Further studies showed that GST activity in the TRV-*SlMYB4* lines (Fig. 7h) and TRV- *SlMYB88* lines (Fig. 7s) was lower than that in the TRV lines. At the same time, ALA rescued the GST activity in the TRV-*SlMYB4* and TRV-*SlMYB88* lines under cold stress. Considering the increase in GST activity, we observed the ROS enrichment in the TRV-*SlMYB4* and TRV*- SlMYB88* lines and determined the ROS content. Our data indicates that ALA decreased the O_2_^.-^ content and accumulation in the leaves of the TRV-*SlMYB4* (Fig. 7f, j) and TRV-*SlMYB88* (Fig. 7i, k) lines, as well as the H_2_O_2_ content and accumulation in the roots of the TRV-*SlMYB4* (Fig. 7q, u) and TRV-*SlMYB88* (Fig. 7t, v) lines. Collectively, these results show that *SlMYB4* and *SlMYB88* were actively involved in the process of improving the cold stress tolerance of tomato via ALA. Overall, ALA promotes *SlGSTU43* expression by upregulating *SlMYB4* and *SlMYB88* expression under cold stress, which improved GST activity, enhanced the ROS-scavenging ability, and improved tomato cold stress tolerance.

## Discussion

Exogenous regulatory substances typically initiate gene expression, regulate metabolic pathways, or activate signaling pathways to achieve specific functions and enhance plant stress tolerance. For instance, by activating *CmRBOHD* expression, melatonin activates Ca^2+^ signaling, which alleviates ABA-induced leaf senescence [15]. γ-aminobutyric acid (GABA) activates ethylene metabolism by increasing the expression levels of *ACC* and *ACS*, thereby enhancing plant resistance under stress conditions [62]. Melatonin slows the senescence of tomato leaves by regulating *SlCV* expression to eliminate excess ROS [66]. As described earlier, ALA plays a crucial role in enhancing plant tolerance to abiotic stress. In tomato, ALA effectively improves tolerance to cold (Fig. 1), NaCl [75], heavy metal [22], and drought stress [45]. Some studies have demonstrated that ALA can reverse stomatal closure caused by ABA in suitable environments for plants [4], and enhances fruit pigmentation through regulating carotenoid metabolism [80]. However, a significant body of research indicates that ALA does not influence plant stress resistance indicators or the capacity to scavenge ROS under suitable conditions for plants [41, 59]. In our previous findings, we discovered that ALA has almost no effect on the content and accumulation of ROS, as well as the cold resistance indices of tomato seedlings under normal temperature conditions (Fig. 1; [33, 73]). The molecular mechanism of ALA improving the cold resistance of tomato is not yet fully understood. A previous multiomic analysis showed that ALA can improve tomato cold tolerance through the regulation of glutathione metabolism and the induction of the expression levels of multiple *SlGSTs* [73].

The present research showed that SlGSTU43 was significantly induced by cold stress at most time points (Fig. 2b). However, at 12 hours, *SlGSTU43* was not significantly induced, and at 48 hours, the expression of *SlGSTU43* was reduced. This finding may be due to the unstable expression of *SlGSTU43* under cold stress, and this instability makes tomato seedlings susceptible to cold stress. Meanwhile, studies have shown that natural factors, such as circadian rhythms, can cause fluctuations in gene expression [16]. In the present study, ALA enhanced *SlGSTU43* expression under cold stress, and *SlGSTU43* was found to be involved in the process by which ALA improved tomato cold tolerance (Fig. 2). SlGSTU43 was localized to the endoplasmic reticulum and plasma membrane (Fig. 3). The role of *SlGSTU43* in tomato's resistance to cold stress was further determined using *SlGSTU43*-overexpressing lines and *slgstu43* mutant lines. The *SlGSTU43*-overexpressing lines showed increased tolerance to cold stress, whereas the *slgstu43* mutant lines were more sensitive to cold stress (Fig. 4). ALA did not improve the cold tolerance of *slgstu43* mutant lines under cold stress (Fig. 5). This discovery confirms that ALA can enhance the cold resistance of tomatoes by upregulating the expression of *SlGSTU43*, and that *SlGSTU43* plays a pivotal role in ALA's mechanism for improving tomato cold resistance.

Research has shown that dwarf phenotypes are closely related to plant adaptability to abiotic stress [13, 30]. Interestingly, the *SlGSTU43*-overexpressing lines showed a dwarf phenotype (Fig. S5) indicating that from a different perspective, the *SlGSTU43*-overexpressing lines may have increased cold tolerance. The dwarfing of plants is closely related to the regulation of endogenous hormones [1, 46]. Overexpression of *GST* in *Phaseolus vulgaris* [48] and *Nicotiana tabacum* [7] induced changes in the endogenous hormone contents of plants. Therefore, the overexpression of *SlGSTU43* in tomato may result in dwarfing via the effects on endogenous hormone content changes. Our future work will focus on elucidating how *SlGSTU43* overexpression causes dwarfing in tomato by altering hormone signaling and metabolism, as well as its role in enhancing cold tolerance.

Excessive accumulation of ROS is one of the main reasons for plant damage caused by cold stress, and timely removal of excess ROS is an effective means to improve plant cold tolerance [26]. Studies have shown that the ROS clearance of chrysanthemum and trifoliate orange was enhanced through upregulated expression of *DgMYB2* [64] and *ERF108* [23], respectively, to improve plant cold stress tolerance. In the present study, ALA increased the GST activity of TRV or TRV-*SlGSTU43* lines to eliminate accumulated ROS, thus improving tomato's cold stress tolerance (Fig. 2g). However, in tomato, when *SlGSTU43* is knocked out, ALA can no longer increase the cold resistance of tomato seedlings by improving GST activity and ROS scavenging ability (Fig. 5e). In tobacco, overexpression of *GhGST* enhanced the plants' ability to eliminate ROS, thus increasing the plant tolerance to *Verticillium* wilt (Li et al., 2018). Overexpression of *H3557* (a *GST*) in cyanobacteria increased the activity of GST, enhanced the ability to remove ROS, and increased tolerance to salt stress [25]. The results of this study showed that ALA can effectively improve the ROS-scavenging ability of tomato by increasing *SlGSTU43* expression, and this improvement depends on the expression of *SlGSTU43* under cold stress, thus improving tomato's cold stress tolerance. A small increase in ROS is often seen as a plant signal inducing and coordinating the plant to strengthen its own defense system [11]. In our previous research, ALA treatment increased the content of H_2_O_2_ in tomato seedling leaves after 12 hours, thus activating the ROS signal of tomato and improving tomato's cold stress tolerance [33]. These findings highlighted the complexity of the functional mechanism through which ALA improves plant cold stress tolerance.

In general, functional protein-coding genes are directly regulated by TFs [14, 58] to function under cold stress or other stresses. In this study, using LUC (Fig. 6e), EMSA (Fig. 6f, g), Y1H (Fig. 6d), and ChIP-qPCR analyses (Fig. 6h, i), it has been comprehensively confirmed that SlMYB4 and SlMYB88 can directly bind to the promoter of *SlGSTU43* to regulate its expression under cold stress. MYB TFs, as one of the largest families in plants, play a crucial role in plant growth, development, and adaptation to various stresses [19, 52]. In this study, LUC (Fig. 6e), EMSA (Fig. 6f, g), Y1H (Fig. 6d), and ChIP-qPCR assays (Fig. 6h, i) comprehensively confirmed that SlMYB4 and SlMYB88 can directly bind to the promoter sequence of *SlGSTU43* to regulate the expression of *SlGSTU43*. In *Senecio cruentus*, ScMYB3 and ScMYB6 can increase the contents of anthocyanins and enhance resistance by activating the ScGST3 promoter [5]. Similarly, PpMYB10.1-activated *PpGST1* plays an important role in peach fruit coloring [79]. Therefore, our research results suggest that SlMYB4 and SlMYB88 can enhance the ROS scavenging ability of tomatoes under cold stress by regulating the expression of *SlGSTU43*.

VIGS was used to prove that *SlMYB4* and *SlMYB88* are involved in the process by which ALA improves tomato cold stress tolerance (Fig. 7). The TRV-*SlMYB4* and TRV-*SlMYB88* lines showed high sensitivity to cold stress, and ALA improved the cold stress tolerance of these lines (Fig. 7a, l). The whole-genome identification of MYB TFs in tomato revealed that SlMYB4 is highly homologous to AtMYB4 in *Arabidopsis* and SlMYB4 is speculated to play a crucial role in nuclear transport [29]. Research has found that overexpression of *SlMYB4* in tomatoes can significantly reduce the expression levels of structural genes such as *SlPAL*, *Sl4CL*, *SlC4H*, and *SlCCR*, thereby reducing the accumulation of lignin [68]. Research on MYB4 and MYB88 in other species indicated that high temperature promote the formation of heterodimers between GhMYB4 and GhMYB66 in cotton, which in turn induces the expression of *Gossypium hirsutum Casein kinase I* [28]. Moreover, MYB4 can affect the flavonoid biosynthesis pathway by repressing the expression of *Arogenate Dehydratase 6* [51]. MdMYB88 can directly up-regulate the expression levels of *COLD SHOCK DOMAIN PROTEIN 3* and *CIRCADIAN CLOCK ASSOCIATED 1* to enhance the cold tolerance of apples [60]. Moreover, the *Arabidopsis myb88flp-1* double mutant is vulnerable to salt and drought stress [61]. In conclusion, SlMYB4 and SlMYB88 are important TFs in tomato's resistance to cold stress. Exogenous regulatory substances, such as melatonin [76], ethylene [69], salicylic acid [65], and proline [73], can influence plant tolerance to abiotic stress, synthesis of substances, and growth and development by modulating the expression of *MYBs*. The data showed that ALA improved the ability of the TRV-*SlMYB4* (Fig. 7f, i-k) and TRV-*SlMYB88* (Fig. 7q, t-v) lines to scavenge ROS under cold stress. In addition, the *SlGSTU43* expression trend in the TRV-*SlMYB4* and TRV-*SlMYB88* lines was consistent with that of *SlMYB4* and *SlMYB88* under different treatments (Fig. 7g, r), indicating that *SlGSTU43* is regulated by SlMYB4 and SlMYB88. Taken together, these results suggest that ALA could regulate *SlGSTU43* expression through the activation of SlMYB4 and SlMYB88 under cold stress, thus improving the ROS scavenging ability of tomato seedlings.

The synthesis and degradation pathways of ALA, which is the unique metabolic precursor of tetrapyrrole biosynthesis in plants, have been characterized [47]. However, when ALA is sprayed onto plants to alleviate stress, it may serve as a plant signal because the existence of plant ALA receptors or elements has not been confirmed, and the specific details in which ALA acts as a plant signaling molecule have not been determined. Therefore, whether ALA could be used as a plant signal and whether ALA receptors exist constitute an important research direction. GABA, which is a nonprotein amino acid, can regulate the drought resistance of *Arabidopsis* by regulating aluminum-activated malate transporters in guard cells, proving that GABA functions as a plant signal [62]. Similarly, NIN-like protein 7 is a nitrate receptor that can regulate nitrogen utilization in *Arabidopsis* by sensing nitrate signals [32]. These findings provide important ideas for future research.

**Figure 8:**
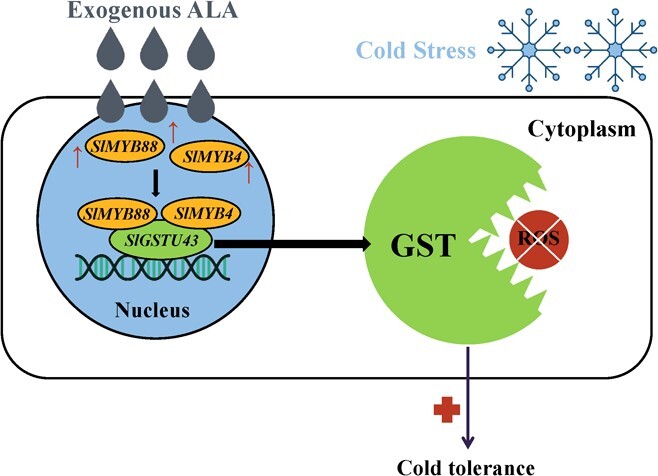
Proposed model for how ALA improves tomato cold tolerance under cold stress. ALA activates *SlMYB4* and *SlMYB88* expression under cold stress. SlMYB4 and SlMYB88 bind to the *SlGSTU43* promoter and activate its expression, which enhances tomato ROS-scavenging ability and ultimately improves tomato cold tolerance.

On the basis of the current findings, a working model for how ALA improves tomato's cold stress tolerance was introduced (Fig. 8). The involvement of *SlGSTU43* in the process of ALA improving tomato's cold stress tolerance was confirmed. The study showed that ALA first activates *SlMYB4* and *SlMYB88* expression levels, and then SlMYB4 and SlMYB88 upregulate *SlGSTU43* expression by binding to the *SlGSTU43* promoter. The upregulated *SlGSTU43* enhances the ability of tomato to remove ROS by increasing GST activity, ultimately improving its tolerance to cold stress. This new finding has not been reported in all previous studies on ALA for improving plant stress tolerance. The present work illustrated the mechanism by which ALA improves tomato cold stress tolerance through the regulation of *SlGSTU43* expression. *SlGSTU43* was added to the cold-responsive gene repository as a valuable gene that could be used to improve tomato cold stress tolerance through genetic engineering.

## Materials and methods

### Tomato growth conditions and ALA treatments

WT tomato (cultivar Ailsa Craig) was used as the background. The cultivation of tomato seedlings and the culture conditions were conducted following our previous research [72, 73]. Tomato seedlings with fully expanded fifth leaves were divided into four treatment groups. One group of tomato was sprayed with 6 mL distilled water containing 0.02% Silwet L-77 (Sigma Aldrich, St. Louis, MO, USA) 12 hours before normal temperature treatment (25°C day/18°C night). The second group of tomato was sprayed with 6 mL 25 mg/L ALA (Sigma Aldrich, St. Louis, MO, USA) solution containing 0.02% Silwet L-77 12 hours before normal temperature treatment (25°C day/18°C night). The third group of tomato was sprayed with 6 mL distilled water containing 0.02% Silwet L-77 12 hours before cold treatment (4°C day/4°C night). The fourth group of tomato was sprayed with 6 mL 25 mg/L ALA solution containing 0.02% Silwet L-77 12 hours before cold treatment (4°C day/4°C night). The other environmental conditions were the same as those of the previous planting environment.

### Cold injury index measurements

The measurement of MDA content followed the methods described by Kong et al. [24], while the measurement of REC ratio followed the methods described by Wang et al. [54]. The Fv/Fm ratio was measured according to the methods of Pérez-Bueno et al. [43] with an Open FluorCam FC 800-O (PSI, Brno, Czech Republic).

### ROS content measurements and histochemical staining

The determination of H_2_O_2_ content (BC3590) and O_2_^.-^ content (BC1290) was performed using relevant detection kits purchased from Solarbio Co., Ltd (Beijing, China). Histochemical staining with DAB and NBT was performed following the methods previously reported by Huang et al. [18] to detect the content of O_2_^.-^ and H_2_O_2_ in tomato leaves. According to our previous method [73, 74], we observed the accumulation of ROS, primarily H_2_O_2_, in the roots of tomato seedlings.

### RNA extraction and qRT–PCR analyses

Total RNA extraction, RNA reverse-transcribed, and qRT-PCR analysis were performed following the methods of Pi et al. (2021). The primers used for gene expression are shown in Supplementary Table S1.

### Genomic DNA extraction

Genomic DNA was extracted from tomato leaves following the methods of Nishizawa-Yokoi and Toki (2021).

### Assay of GUS activity

A 1747-bp promoter fragment of *SlGSTU43* was amplified and inserted upstream of the *GUS*, resulting in the generation of pBI121-*SlGSTU43*. pBI121-*SlGSTU43* vector was transformed into tobacco (*Nicotiana benthamiana*) seedlings, following the protocol described by Dai et al. [6]. The transformed tobacco seedlings were divided into three groups: the first group was subjected to normal temperature (25°C day/18°C night) treatment for 1 day, the second group was exposed to cold stress (4°C day/4°C night) for 1 day, and the third group was sprayed with 25 mg·L ALA and then subjected to cold stress (4°C day/4°C night) treatment for 1 day. The *GUS* expression level and GUS staining were determined using the method described by Zhao et al. [78]. The primer sequences used for constructing the pBI121-*SlGSTU43* vector and for *GUS* gene expression analysis can be found in Supplementary Table S2 and Supplementary Table S3, respectively.

### VIGS and experiments

The VIGS system using tobacco rattle virus (TRV) was employed to silence the expression of the *SlGSTU43* gene in tomato. A 248-bp fragment (selected by the use of the SGN VIGS Tool, https://vigs.solgenomics.net/) of *SlGSTU43* was amplified and inserted into the pTRV2 vector to generate a pTRV2-*SlGSTU43* construct. The pTRV1, pTRV2, and pTRV2-SlGSTU43 constructs were individually transformed into *Agrobacterium tumefaciens* strain GV3101 (pSoup-p19). The transformed *A. tumefaciens* strain GV3101 were injected into the cotyledons of tomato seedlings following the method described by Ekengren et al. [9]. After silencing tomato *SlPDS* (XM_010320112.2) via VIGS, the tomato seedling leaves showed a photobleached phenotype [35]. Accordingly, a pTRV2-*SlPDS* vector was used as the control (Fig. S1a). pTRV2-*SlPDS*, pTRV2-*SlMYB4*, and pTRV2-*SlMYB88* were constructed in the same way as was pTRV2-*SlGSTU43*. The primers used for VIGS vector construction are shown in Supplementary Table S2.

The TRV lines were divided into three treatment groups, following the same protocols as described earlier. After 6 days of treatment, tomato leaves and roots were obtained for physiological measurements and histochemical staining.

### GST activity measurement

GST activity (BC0350) was measured by using relevant detection kits purchased from Solarbio Co., Ltd. (Beijing, China).

### Sequence and promoter cis-acting elements analyses of *SlGSTU43*

The phylogenetic analysis, conservative motif analysis, and tertiary structure prediction of SlGSTU43 were performed according to our previous research [70]. The sequence numbers of the proteins and genes used in the analysis are listed separately in Supplementary Tables S4 andS5. We applied the same method as previously described to analyse the cis-acting elements in the promoter region of *SlGSTU43* [70].

### Relative level analysis of *SlGSTU43* in different tomato tissues

In total, 30 plump tomato seeds were selected as samples. When the fifth leaf of each seedling was fully unfolded, the fourth leaf and the root system, as well as the stem between the second and third leaf, were sampled. Completely open flowers were selected. After removing the seeds, the ripe tomato fruits were homogenized and sampled.

### Subcellular localization analysis

The coding region of *SlGSTU43* without stop codons (TAA) was amplified from cDNA templates of tomato and inserted together with a green fluorescent protein (GFP) in a pCAMBIA2300 vector to generate pCAMBIA2300-*SlGSTU43*. The pCAMBIA2300-*SlGSTU43* fusion construct and the control vector pCAMBIA2300 were then transformed into tobacco leaves. The primers used for pCAMBIA2300-*SlGSTU43* vector construction are shown in Supplementary Table S2. The protein expression was recorded using an Olympus IX83 confocal laser microscope (Tokyo, Japan). The images were arranged and combined using Olympus Fluoview and Adobe Illustrator software.

### Plant transformation and cold treatment

For the *SlGSTU43*-overexpressing lines, full-length *SlGSTU43* was cloned into a pHELLSGATE8 plant expression vector*.* We used CRISPR/Cas9 technology to generate *slgstu43* mutant lines. The binary vector pYLCRISPR/Cas9P35S-N and two helper plasmids (PYLsgRNA-LacZ-AtU3d), (PYLsgRNA-LacZ-AtU3b) [53] were used to generate the CRISPR/Cas9 construct. According to the description by Fillatti et al. [12], transgenic tomato lines were obtained by introducing the constructed vector into the Ailsa Craig using the *Agrobacterium*-mediated transformation method.

The breeding process and culture conditions were the same as those previously described. The *SlGSTU43*-overexpressing lines and WT lines were subjected to cold treatment (4°C day/4°C night) together for 5 days. Similarly, the *slgstu43* mutant lines and WT lines were treated together under cold treatment (4°C day/4°C night) for 3 days. Afterwards, their phenotypes were observed, and physiological indices were measured.

To carry out ChIP assays, we produced *SlMYB4*-overexpressing lines and *SlMYB88*-overexpressing lines. Full-length *SlMYB4* and *SlMYB88* were cloned into a pCAMBIA1300 plant expression vector together with a FLAG tag. The methods of transferring the constructed vector into tomato were the same as those previously described.

### Y1H assays

The promoter fragment (P1) was inserted into a pAbAi vector as bait. Full-length *SlMYB4* and *SlMYB88* were cloned into the vector pGADT7, which served as prey. The bait and prey were cotransformed into cells of the Y1H Gold strain. The primers used for pAbAi and pGADT7 vectors construction are shown in Supplementary Table S2.

### LUC assays

The promoter fragment (P2) of *SlGSTU43* was recombined into a pGreen 0800-LUC vector to generate a reporter. The coding DNA sequences (CDSs) of *SlMYB4* and *SlMYB88* were inserted into a pGreen 62-SK vector to generate two effectors (SK-SlMYB4 and SK-SlMYB88). The tobacco transformation and LUC analysis methods were consistent with those previously described by Zhang et al [71]. The primers used for LUC assays vector construction are shown in Supplementary Table S2.

### EMSA assays

Full-length *SlMYB4* and *SlMYB88* were cloned and ligated into the vector pMAL-c5X. Then, we transformed the recombinant plasmid into *Escherichia coli* strain BL21 (DE3). The subsequent protein induction and purification was performed according to the amylose resin (New England BioLabs, Beijing, China) manufacturer's protocol. The probe labeled and EMSA experiments were conducted according to the manufacturer's protocol (Beyotime, Shanghai, China). The primers used for vector construction are shown in Supplementary Table S2.

### ChIP-qPCR assays

*SlMYB4*-overexpressing and *SlMYB88*-overexpressing lines were subjected to 4°C for 24 hours, and then 1 g of leaf tissue was collected for ChIP assays. The enriched protein was incubated with anti-FLAG antibody (Abmart, Shanghai, China) or actin antibody (Abmart, Shanghai, China). ChIP assays were performed as described by Wang et al. [53]. The obtained DNA product was measured via qRT-PCR together with the specific primers listed in Table S6.

### Statistical analysis

All the experiments were repeated three times for each experiment. Statistical analysis of the bioassays was performed using the SAS software version 8.0 (SAS Institute, Cary, NC, USA) through the Tukey's test at a level of *P* < 0.05.

## Supplementary Material

Web_Material_uhae026
