# Supplementary material for: 5-Aminolevulinic acid improves cold resistance through regulation of SlMYB4/SlMYB88-SlGSTU43 module to scavenge reactive oxygen species in tomato
Source: Hortic Res. 2024 Jan 19;11(3):uhae026. doi: 10.1093/hr/uhae026 (PMC10940124; doi:10.1093/hr/uhae026)
Supplement: Web_Material_uhae026 [file web_material_uhae026.zip › Supplemental Material 1.docx]

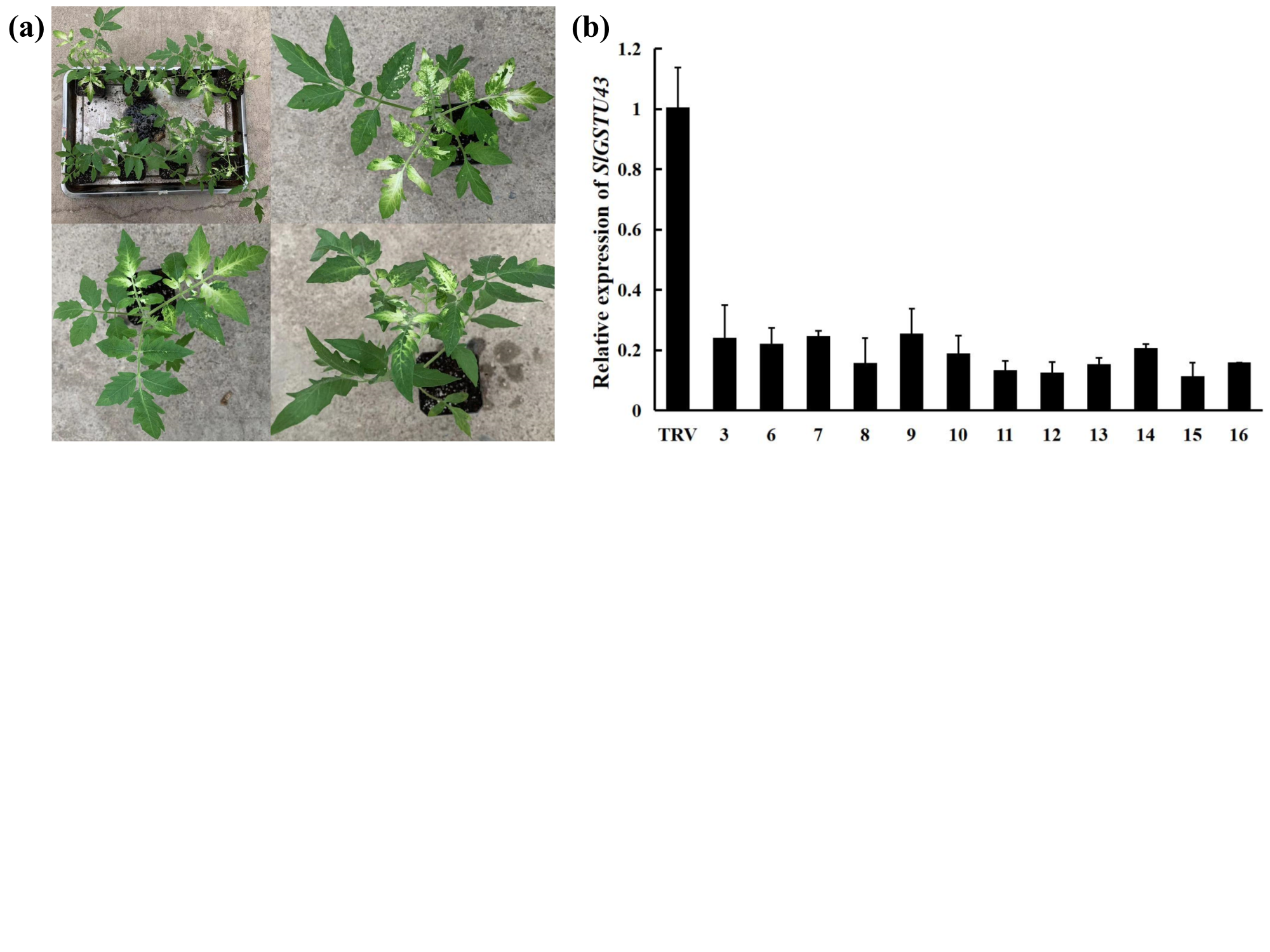


**Fig S1. Phenotype of tomato plants after silencing *SlPDS* with VIGS and screening of TRV-*SlGSTU43* lines.** (a) After silencing tomato *phytoene desaturase* (*SlPDS,* XM_010320112.2) gene with VIGS, tomato seedling leaves showed photo-bleaching phenotype. (b) qRT**-**PCR was used to analyze the *SlGSTU43* expression of TRV2-*SlGSTU43* lines. The error bars represent ± SDs (*n* = 3).


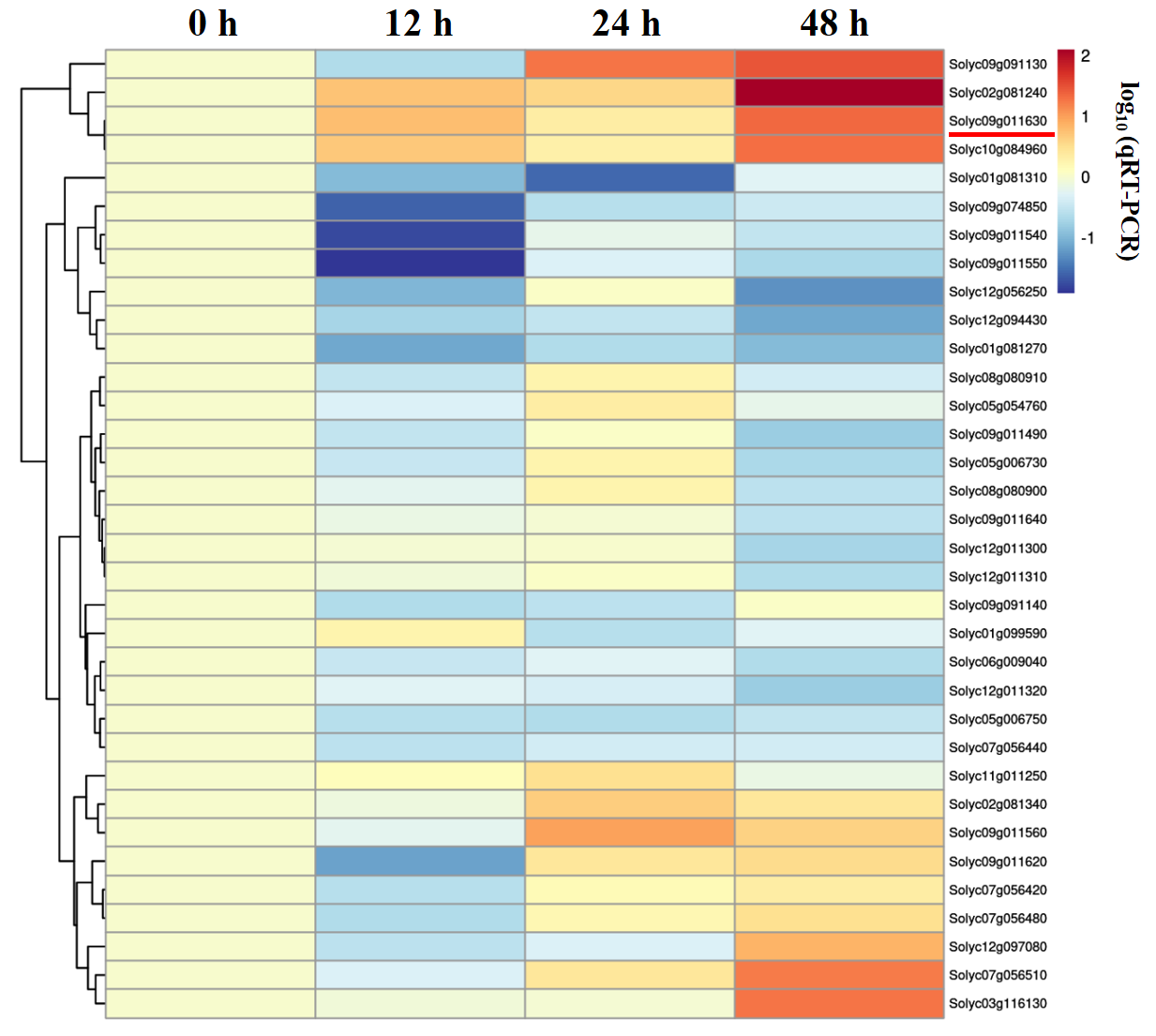


**Fig S2. Expression profiles of 34 *SlGSTs* induced by ALA in tomato seedling leaves under cold stress (4°C day/4°C night, 12 h/12 h) for 0, 12, 24, and 48 h.** The red font indicates the target gene (SlGSTU43, Solyc09g011630). The concentration of ALA used was 25 mg·L^−1^.


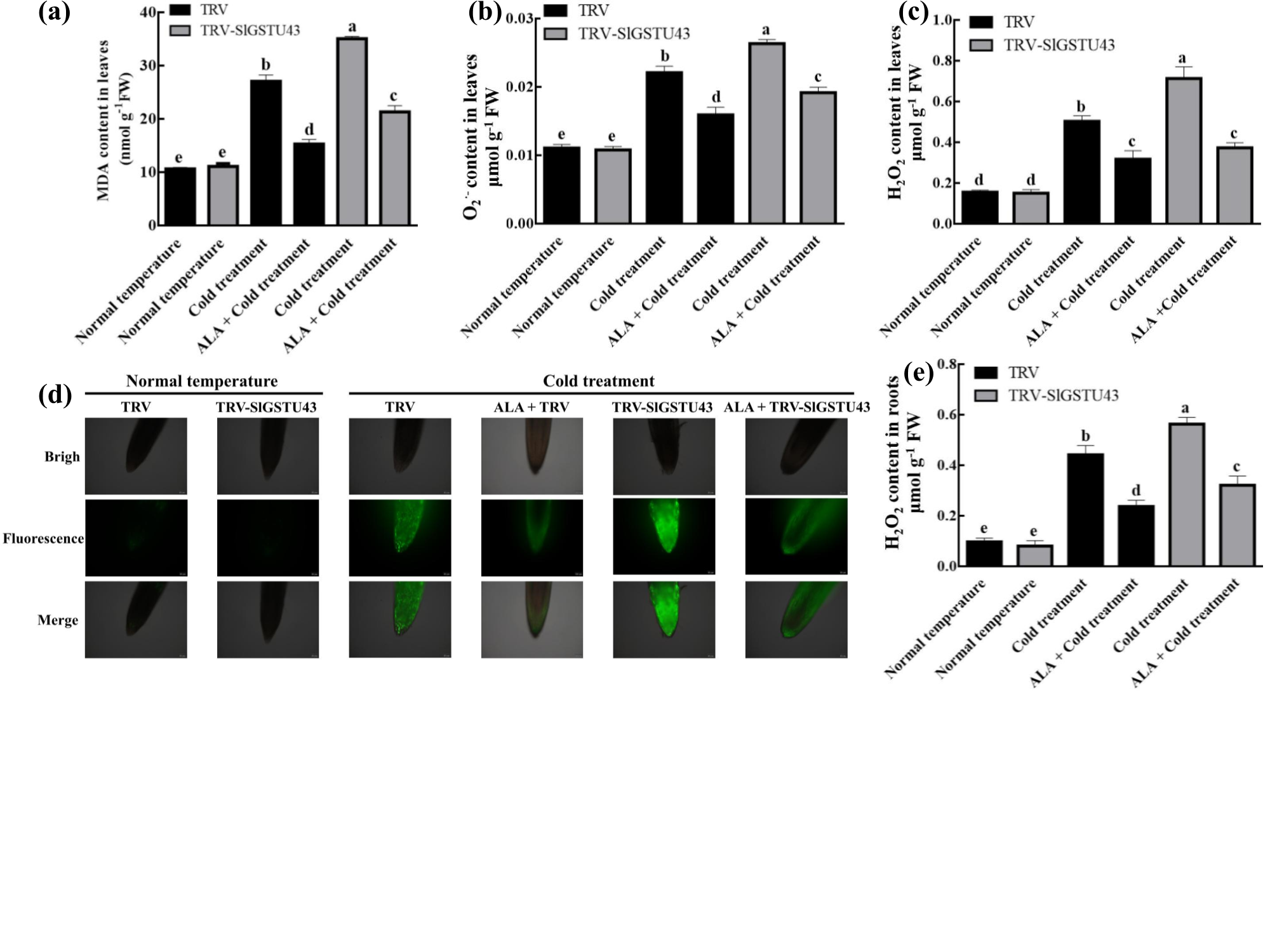


**Fig S3. The effects of ALA on the MDA and ROS content in TRV and TRV-*SlGSTU43* lines under cold stress.** (a) MDA content, (b) O_2_^.-^ content, and (c) H_2_O_2_ content in leaves of TRV and TRV-*SlGSTU43* lines. (d) Histochemical staining and (e) H_2_O_2_ content in roots of TRV and TRV-*SlGSTU43* lines. All the indexes of the tomato seedlings were obtained after exposure to normal temperature (25°C day/18°C night, 12 h/12 h) and cold stress (4°C day/4°C night, 12 h/12 h) with or without 25 mg·L^−1^ ALA for 6 days. H_2_O_2_ accumulation was observed with DCFH-DA probe. The error bars represent ± SDs (*n* = 3). The different letters indicate significant differences (*P* < 0.05) according to Tukey’s test.


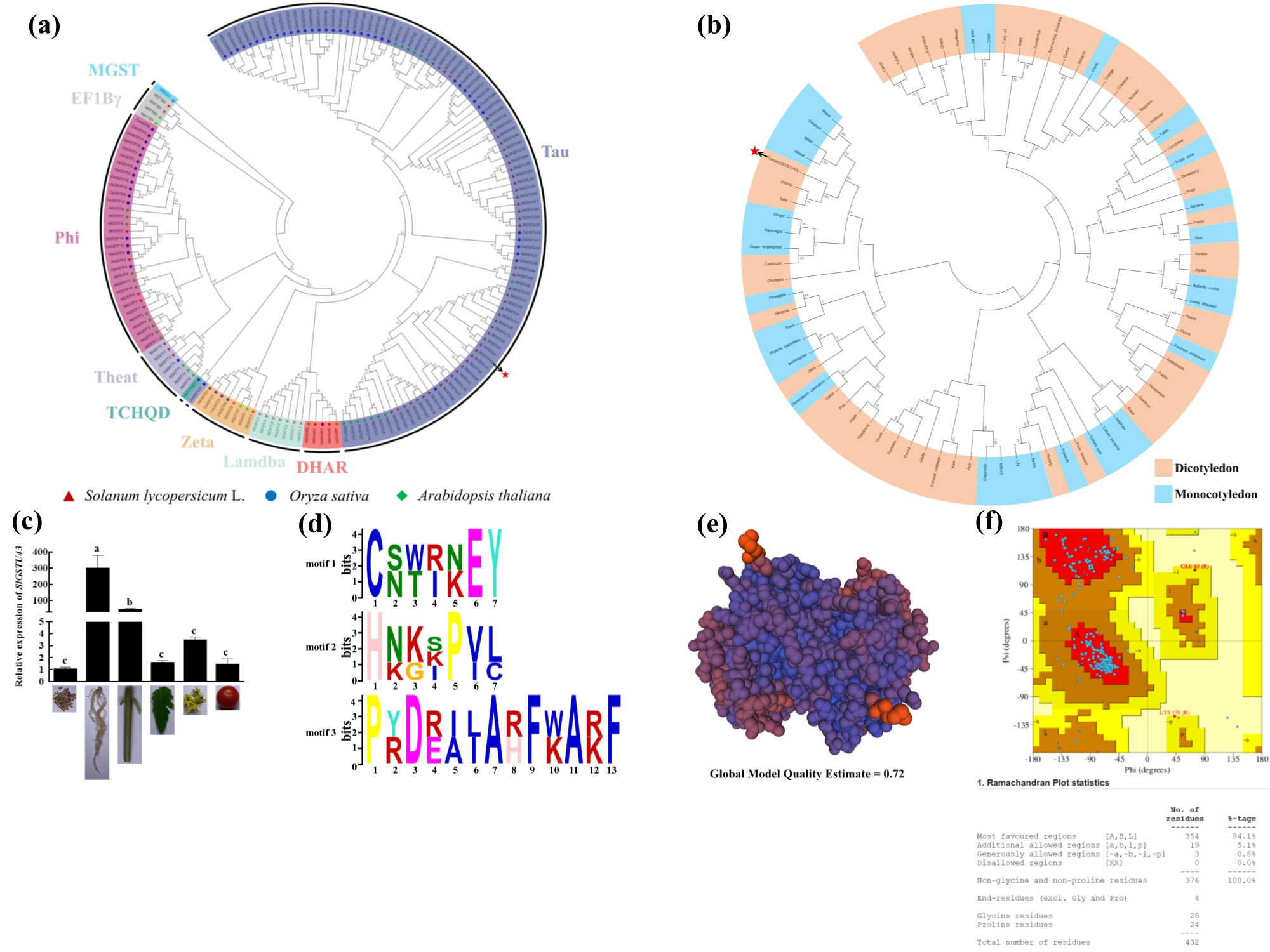


**Fig S4. Characteristic analysis of *SlGSTU43*.** (a) 69 tomato GST proteins, 62 rice GST proteins, and 35 *Arabidopsis thaliana* GST proteins to construct phylogenetic tree. The red pentagram is labeled as our target gene (*SlGSTU43*). (b) 31 monocotyledon *GSTs* and 49 dicotyledonous *GSTs* to construct phylogenetic tree.The red pentagram is labeled as our target gene (*SlGSTU43*). (c) The expression level of *SlGSTU43* in different tissues of tomato. (d) Length and amino acid species of the three conserved motifs of the SlGSTU43 protein sequence. (e) Modeling the tertiary structure of SlGSTU43 protein. (f) Ramachandran plot to assess SlGSTU43 protein tertiary structure. Ramachandran plot can classify the distribution of protein amino acid residues into four criteria: most favored regions (red regions), additional allowed regions (yellow regions), generously allowed regions (light yellow regions) and disallowed regions (white regions). All amino acid residues of SlGSTU43 protein are distributed in most favored regions, additional allowed regions, and generously allowed regions, of which 94.1% are in most favored regions, 5.1% are in additional allowed regions, and 0.8% are in generously allowed regions. Therefore, we speculate that the protein has good structural quality. The error bars represent ± SDs (*n* = 3). The different letters indicate significant differences (*P* < 0.05) according to Tukey’s test.


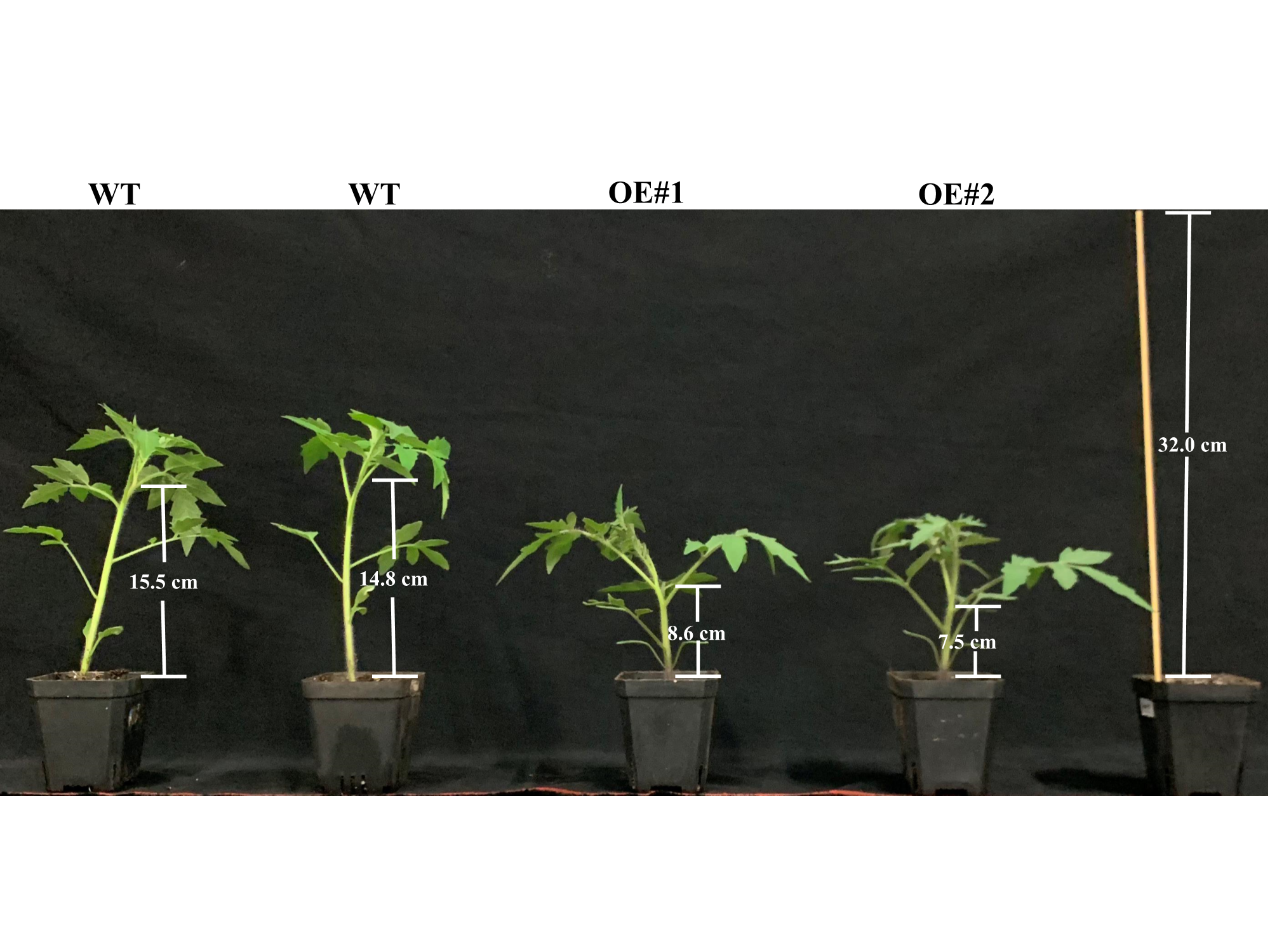


**Fig. S5.** Compared to WT plants, *SlGSTU43*-overexpressing lines show dwarfing.


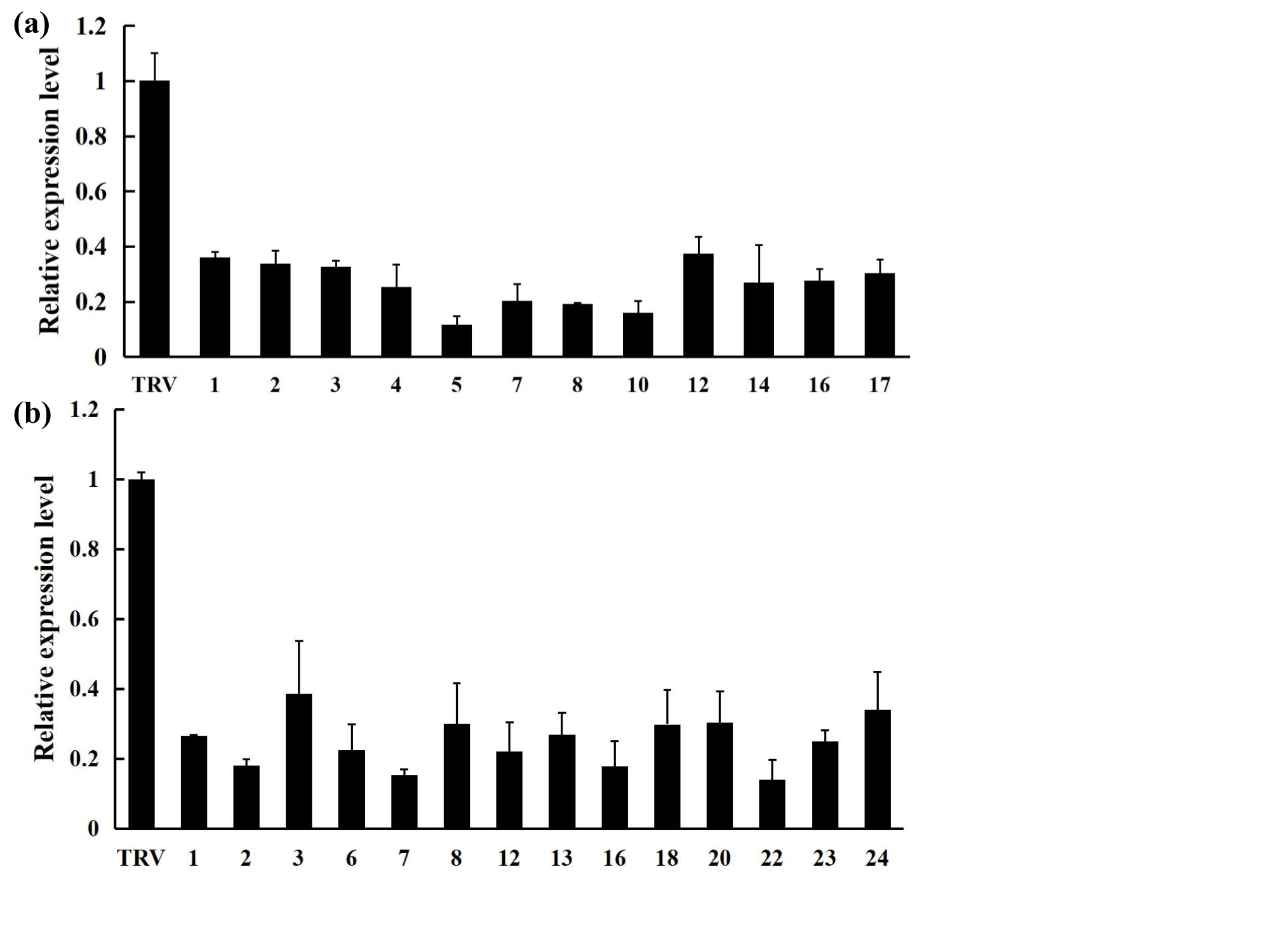


**Fig S6.** Molecular identification of VIGS tomatoes. (a) qRT**-**PCR was used to analyze the *SlMYB4* expression of TRV2-*SlMYB4* lines. (b) qRT**-**PCR was used to analyze the *SlMYB88* expression of TRV2-*SlMYB88* lines. The error bars represent ± SDs (*n* = 3).
