# Supplementary material for: 5-Aminolevulinic acid improves cold resistance through regulation of SlMYB4/SlMYB88-SlGSTU43 module to scavenge reactive oxygen species in tomato
Source: Hortic Res. 2024 Jan 19;11(3):uhae026. doi: 10.1093/hr/uhae026 (PMC10940124; doi:10.1093/hr/uhae026)
Supplement: Web_Material_uhae026 [file web_material_uhae026.zip › Supplemental Material 2.docx]

**Table S1** List of primer sequences used for qRT-PCR analysis.

| **Gene ID** | **Forward primer** | **Reverse primer** |
| --- | --- | --- |
| **Solyc09g011630 (*SlGSTU43*)**  NM_001321306.1 (*Actin7)*  Solyc09g090130 (*SlMYB4*)  Solyc05g007160 (*SlMYB88*)  Solyc09g074850  Solyc02g081340  Solyc12g094430  Solyc06g009040  Solyc08g080900  Solyc08g080910  Solyc12g056250  Solyc05g054760  Solyc11g011250  Solyc09g011490  Solyc09g011540  Solyc09g011550  Solyc09g011560  Solyc09g011620  Solyc09g011640  Solyc01g081270  Solyc09g091130  Solyc12g011300  Solyc12g011310  Solyc12g011320  Solyc05g006730  Solyc05g006750  Solyc12g097080  Solyc07g056420  Solyc07g056440  Solyc07g056480  Solyc07g056510  Solyc09g091140  Solyc10g084960  Solyc01g081310  Solyc03g116130  Solyc02g081240  Solyc01g099590  Solyc01g081250  Solyc06g075520  Solyc07g056460  Solyc07g056500  Solyc09g011600  Solyc09g011650  Solyc09g011510  Solyc09g011520  Solyc09g011590  Solyc02g083630  Solyc09g007270  Solyc09g075900  Solyc04g012060  Solyc01g100960 | TCTCAAGATTAAGGGTGTT  TGTCCCTATTTACGAGGGTTATGC  GCTTGGCAATAGATGGT  CAACAAGGCAATGTAGAAG  TGGATGGAAGTAGAAGGC  AGGGAAAGAAACTAACCG  GGAAGTCAAGGTAACAAGGG  GCTGACAAGGGGAACCAACT  ATACTGTTCTTGCTCCTGC  ATGGGATAGACTTTGAGG  GGTCAACATCGCACTCCT  TTTCCTACCTTCGTCTCATTTC  CTGGTTTTTGAAGATAAGCCC  GGCAAGCCCATTTGTGAG  GCTCTCAAGATTAAGGGTG  GCTTGGGCTATGGTATA  TTGGTGTTAGTGGCAGTTC  TCTCAAGATTAAGGGTGTCC  GCTCGTTTTTGGGCTAAG  GCTTTTGTTCCAGCAGTG  CATGATGGAAAACCAAT  GTATGTTTGGTATGAGGG  GTATGTTTGGTATGAGGG  GGGAGTCAATTTCATCC  CATGATGGAAAACCTATT  GCGTTGAAATAGCCCTC  TGAATACATCAACGAGAG  GCATGTATGGTATGAGGCTAAG  CATAACGGGAAACCAAT  AGGTTTTGGGCTGATTAC  TGCTTGATTTATGGGTTAG  GTTTACTTATAGGGTTATTTGG  CTTGGTGTTTATGGGAG  AGTTGTATGATTTCGGGAG  TTCCGCTACTCCTTCAC  GAAGACTTATTTGGTGG  TTGTGCTTATGGACGCTC  GGATGGCGTATCTTCTTGATGTAT  GTACTGAGCAGGCACTTTTTGAT  AGCAGGAGATAGCGAAGAAAGAT  TAGTGATCCTTACAAGAGAGCTCAT  TCTCGAATACATCGACGAGACAT  AAGGGAAATCCAATTCCTGAGT  AGGCAAAGGAGAGGAGAATGAG  GGTTGAGTGGGCTCTAAAGAT  TTAAGGGAGTGAAATATGAATTTAT  GTTCAGTGCTCCTGATTTCCTT  CCTATCCTACGCTGACTTTTATCAG  GAGGAGGTGAAGGTTCTACAGC  AAGGTCTTGCAGATACATTCATATT  TATTACCTACCGTTATTGTCCATTC | GGCAAGATGGAAGGAC  CAGTTAAATCACGACCAGCAAGAT  GCTTTGAGGAGGCTGAT  ATCTGAAATGTCGCTCC  CGAGTTTCGTAGATGTCAAG  TTTAGGGAATACGAGGAG  GCTTTGTCCTTCTGCCTC  TTCTTCGTTCTCCGCCAC  CCATAATCTCACACACTAAGC  ATAGCTGGTATTTGCCG  GACACATTCTACATTTGCTC  CAACTGAACAAACATTCTGCCC  AGTTGTCAGCGGAGGTTTG  TGCTCCCACCTTATCATCG  TTAGGCAAGATGGAAGG  AACACGATCATAAGGGTC  GAGGGCTTTTGTTTTGTAG  GGGCCTTCAAATGTTTC  CAACAACTCACCACACTCCT  AAGATACGCCATCCAACC  CAAAACCTAGCCAAAGC  TCACAAATTGGTTTTCC  TCACAAATTGGTTTTCC  CACAAATAGATTTACCCTTG  TGCTGGAAACCACTTAT  CTCGTCGTGGAACAGAAC  GAGATGTGGCTCGTTC  TGTTCCTCAAATCCTCGTC  TTGTCAATGTAATCAACCC  CCAAGTGCTCCCTCTAAC  TGCTTATGGATAGGGTT  CTTCTATATACTCAAATGGTATTC  GATTTAAGAAGTAAAGGGC  TAAGGCTTGTCTCCCAGT  TTCATAGGGATCTTCTGG  CAACAAAGGGTTCGTAG  CATACCCAAAGTTGTCTCCTC  GTTACTAGTTTGTCTCTGGGTGGC  TGAGTCAAGCTTTCAGTCACACTC  TGAAGTTACCGTAAGTCTCATAGGC  AATTCACCTTCTAACACTTTCAAAC  TCATCGAAGAATTTAGCCCAGA  AATTGCTGTCATAACAAAGTTTCAG  CGACTCCAGAGCCTTCTTGG  TCTCGTCTATGTACTCAAGAATGAC  CAAATGCCTCATCAATGTATTC  GATACGTTATTATGGGATGATTGG  CAATGTCTTTATCACTCAATCCCAT  GCTTCTTTATTAACCTCCAAGTATG  CTGGACAATACCCTTGCTTACT  TTCCAGTTCCTTCAACATTAAAAG |

**Table S2** List of primer sequences used for vector construction.

| **Vector** | **primer** |
| --- | --- |
| pBI121-*SlGSTU43*  pTRV2-*SlGSTU43*  pTRV2-*SlMYB4*  pTRV2-*SlMYB88*  pTRV2-*SlPDS*  pCAMBIA2300-*SlGSTU43*  pHellsgate8-*SlGSTU43*  AtU3bT1-*SlGSTU43*  AtU3dT2-*SlGSTU43*  pCAMBIA1300-*SlMYB4*  pCAMBIA1300-*SlMYB88*  pGreen 62-SK-*SlMYB4*  pGreen 62-SK-*SlMYB88*  pGreen0800-LUC  -*SlGSTU43*  pMAL-c5X-*SlMYB4*  pMAL-c5X-*SlMYB88*  pGADT7-*SlMYB4*  pGADT7-*SlMYB88*  pAbAi-*SlGSTU43* | **Forward**: gaccatgattacgccaagcttTTTGGTCAAAAATATTATTTAAC  **Reverse**: ggactgaccacccggggatccTTTGGTTTTCTTTTATAGATG  **Forward**: aaggttaccgaattctctagaTTTGATGATAAGTGC  **Reverse**: cgtgagctcggtaccggatccTAAATATTCCTTGATTG  **Forward**: aaggttaccgaattctctagaGTATGGCACACTCACTTGAAAAAAA  **Reverse**: cgtgagctcggtaccggatccGAGATGTCCATTGTCACGGCTAG  **Forward**: aaggttaccgaattctctagaATGCAGAGTGGTATG  **Reverse**: cgtgagctcggtaccggatccAAAAATCTTTTGAGCC  **Forward**: aaggttaccgaattctctagaATGCCTCAAATTGGACTTGTTTCTG  **Reverse**: cgtgagctcggtaccggatccCTAAACTACGCTTGCTTCCGACAAC  **Forward**: cgggggacgagctcggtaccATGGCAGGAGTAAAGTTGCTA  **Reverse**: accatggtgtcgactctagaTTTTTGGGCAGCTGCAAAAC  **Forward**:catttggagaggacacgctcgagATGGCAGGAGTAAAGTTGCTA  **Reverse**:tctcattaaagcaggactctagaTTTTTGGGCAGCTGCAAAAC  **Forward**:GTAATGGGGAAAGCTATATTTGGgttttagagctagaaat  **Reverse**:CCAAATATAGCTTTCCCCATTACtgaccaatgttgctcc  **Forward**:GATTTGATGGCATATTGGATGGGgttttagagctagaaat  **Reverse**:CCCATCCAATATGCCATCAAATCtgaccaatgttgctcc  **Forward**:cagtggtctcactctATGGTTAGAGCTCCTTGTTGT  **Reverse**:cagtggtctcactgcAAATTCTGGTAACTCTAATAAGTCC  **Forward**:cagtggtctcactctATGCAGAGTGGTATGAAGAAAAG  **Reverse**:cagtggtctcactgcTAGACTGTTGAGGAGGGCTC  **Forward**:cgcggtggcggccgctctagaATGGTTAGAGCTCCTTG  **Reverse**:gtcgacggtatcgataagcttTCAAAATTCTGGTAACTC  **Forward**:cgcggtggcggccgctctagaATGCAGAGTGGTATGAAGAAAAG  **Reverse**:gtcgacggtatcgataagcttTTATAGACTGTTGAGGAGGGCTC  **Forward**:ctatagggcgaattgggtaccCCGAAAACATCCTTTAAGTATTTAT  **Reverse**:caggaatcgatacaagcttGTTTCTTCTCAATTGAATATGCTAG  **Forward**:gagggaaggatttcacatatgATGGTTAGAGCTCCTTG  **Reverse**: cctgcagggaattcggatccTCAAAATTCTGGTAACTC  **Forward**:gagggaaggatttcacatatgATGCAGAGTGGTATGAAGAAAAG  **Reverse**:cctgcagggaattcggatccTTATAGACTGTTGAGGAGGGCTC  **Forward**:gccatggaggccagtgaattcATGGTTAGAGCTCCTTG  **Reverse**:agctcgagctcgatggatccTCAAAATTCTGGTAACTC  **Forward**:gccatggaggccagtgaattcATGCAGAGTGGTATGAAGAAAAG  **Reverse**:agctcgagctcgatggatccTAGACTGTTGAGGAGGGCTC  **Forward**:cttgaattcgagctcggtaccGTGTGAGCGTACATGATTTG  **Reverse**:agcacatgcctcgaggtcgacCGTAATTGAGTTAAGAATTATGC |

**Table S3** List of primer sequences used for *GUS* gene expression.

| Application | **primer** |
| --- | --- |
| GUS qRT-PCR  qRT-PCR reference gene (α-tubulin) | **Forward**: GATCGCGAAAACTGTGGAAT  **Reverse**: TAATGAGTGACCGCATCGA  **Forward**: ATGAGAGAGTGCATATCGAT  **Reverse**: TTCACTGAAGAAGGTGTTGAA |

**Table S4** List of protein sequences used to construct phylogenetic tree.

| Species | Proteine name | Protein ID | Proteine name | Protein ID |
| --- | --- | --- | --- | --- |
| *Solanum lycopersicum* L. | SlDHAR1 | Solyc05g054760 | SlDHAR2 | Solyc11g011250 |
|  | SlEF1Bγ1 | Solyc11g028100 | SlEF1Bγ2 | Solyc06g011280 |
|  | SlGSTF1 | Solyc09g074850 | SlGSTF2 | Solyc05g006750 |
|  | SlGSTF3 | Solyc06g009020 | SlGSTF4 | Solyc12g094430 |
|  | SlGSTF5 | Solyc06g009040 | SlGSTL1 | Solyc04g009530 |
|  | SlGSTL2 | Solyc09g007150 | SlGSTL3 | Solyc10g084400 |
|  | SlGSTL4 | Solyc12g044520 | SlGSTL5 | Solyc12g044530 |
|  | SlGSTL6 | Solyc00g007030 | SlGSTL7 | Solyc00g007040 |
|  | SlGSTT1 | Solyc08g080900 | SlGSTT2 | Solyc08g080910 |
|  | SlGSTT3 | Solyc12g056250 | SlGSTU1 | Solyc12g036560 |
|  | SlGSTU2 | Solyc09g063150 | SlGSTU3 | Solyc09g011490 |
|  | SlGSTU4 | Solyc09g011500 | SlGSTU5 | Solyc09g011520 |
|  | SlGSTU6 | Solyc09g011540 | SlGSTU7 | Solyc09g011550 |
|  | SlGSTU8 | Solyc09g011560 | SlGSTU9 | Solyc09g011570 |
|  | SlGSTU10 | Solyc09g011580 | SlGSTU11 | Solyc09g011590 |
|  | SlGSTU12 | Solyc09g011600 | SlGSTU13 | Solyc09g011620 |
|  | SlGSTU15 | Solyc09g011640 | SlGSTU16 | Solyc01g081260 |
|  | SlGSTU17 | Solyc01g081270 | SlGSTU18 | Solyc09g091130 |
|  | SlGSTU19 | Solyc12g011300 | SlGSTU20 | Solyc12g011310 |
|  | SlGSTU21 | Solyc12g011320 | SlGSTU22 | Solyc05g006730 |
|  | SlGSTU23 | Solyc05g006740 | SlGSTU24 | Solyc05g006750 |
|  | SlGSTU25 | Solyc05g026210 | SlGSTU26 | Solyc12g097080 |
|  | SlGSTU27 | Solyc07g056420 | SlGSTU28 | Solyc07g056430 |
|  | SlGSTU29 | Solyc07g056440 | SlGSTU30 | Solyc07g056450 |
|  | SlGSTU31 | Solyc07g056460 | SlGSTU32 | Solyc07g056470 |
|  | SlGSTU33 | Solyc07g056480 | SlGSTU34 | Solyc07g056490 |
|  | SlGSTU35 | Solyc07g056500 | SlGSTU36 | Solyc07g056510 |
|  | SlGSTU37 | Solyc09g091140 | SlGSTU38 | Solyc10g084960 |
|  | SlGSTU40 | Solyc10g007620 | SlGSTU41 | Solyc10g007640 |
|  | SlGSTU42 | Solyc01g081310 | SlGSTU14 | Solyc06g069040 |
|  | SlGSTU43 | Solyc09g011630 | SlGSTU44 | Solyc03g116130 |
|  | SlGSTU45 | Solyc03g116120 | SlGSTU46 | Solyc02g081240 |
|  | SlGSTU47 | Solyc01g099590 | SlGSTU48 | Solyc01g086680 |
|  | SlGSTZ1 | Solyc01g091330 | SlGSTZ2 | Solyc01g102660 |
|  | SlMGST | Solyc04g081740 | SlDHAR1 | Solyc05g054760 |
|  | SlDHAR2 | Solyc11g011250 | SlEF1Bγ1 | Solyc11g028100 |
|  | SlEF1Bγ2 | Solyc06g011280 |  |  |
| *Arabidopsis thaliana* | AtGSTU1 | AT2G29460 | AtGSTU2 | AT1G69920 |
|  | AtGSTU3 | AT2G29470 | AtGSTU4 | AT1G53680 |
|  | AtGSTU5 | AT1G69920 | AtGSTU6 | AT2G29440 |
|  | AtGSTU7 | AT2G29450 | AtGSTU8 | AT2G29490 |
|  | AtGSTU9 | AT1G74590 | AtGSTU10 | AT1G27140 |
|  | AtGSTU11 | AT1G59700 | AtGSTU12 | AT1G59670 |
|  | AtGSTU13 | AT1G10360 | AtGSTU14 | AT2G29480 |
|  | AtGSTU15 | AT2G29420 | AtGSTF1 | AT1G02930 |
|  | AtGSTF2 | AT1G02920 | AtGSTF3 | AT2G02930 |
|  | AtGSTF4 | AT4G02520 | AtGSTF5 | AT2G47730 |
|  | AtGSTF6 | AT5G17220 | AtGSTF7 | AT3G03190 |
|  | AtGSTF8 | AT3G62760 | AtGSTF9 | AT1G49860 |
|  | AtGSTF10 | AT1G02950 | AtGSTF11 | AT1G02940 |
|  | AtEF1Bγ1 | AT1G57720 | AtEF1Bγ2 | AT1G09640 |
|  | AtGSTT1 | AT5G41210 | AtGSTT2 | AT5G41240 |
|  | AtGSTT3 | AT5G41220 | AtDHAR1 | AT5G16710 |
|  | AtDHAR2 | AT1G19570 | AtGSTZ1 | AT2G02390 |
|  | AtGSTZ2 | AT2G02380 |  |  |
| *Oryza sativa* | OsGSTU1 | Os03g0785900 | OsGSTU2 | Os10t0529800 |
|  | OsGSTU3 | Os07t0468100 | OsGSTU4 | Os10t0528400 |
|  | OsGSTU5 | Os09t0367700 | OsGSTU6 | Os01t0558100 |
|  | OsGSTU7 | Os01t0949700 | OsGSTU8 | Os10t0529700 |
|  | OsGSTU9 | Os10t0529400 | OsGSTU10 | Os10t0531400 |
|  | OsGSTU11 | Os07t0168300 | OsGSTU12 | Os10t0527800 |
|  | OsGSTU14 | Os10t0528900 | OsGSTU15 | Os10t0525400 |
|  | OsGSTU16 | Os05t0412800 | OsGSTU17 | Os09t0467200 |
|  | OsGSTU18 | Os10t0528200 | OsGSTU19 | Os10t0527400 |
|  | OsGSTU20 | Os10t0527601 | OsGSTU21 | Os10t0525500 |
|  | OsGSTU22 | Os10t0530400 | OsGSTU23 | Os10t0528100 |
|  | OsGSTU24 | Os10t0530200 | OsGSTU25 | Os10t0530300 |
|  | OsGSTU26 | Os10t0529500 | OsGSTU27 | Os10t0525400 |
|  | OsGSTU28 | Os10t0365200 | OsGSTU29 | Os10t0395400 |
|  | OsGSTU30 | Os01t0949800 | OsGSTU31 | Os06t0227500 |
|  | OsGSTU32 | Os01t0692100 | OsGSTU33 | Os01t0950000 |
|  | OsGSTU34 | Os03t0643700 | OsGSTU35 | Os07t0152700 |
|  | OsGSTU36 | Os07t0468100 | OsGSTU37 | Os10t0481300 |
|  | OsGSTU38 | Os10t0530600 | OsGSTU39 | Os10t0530900 |
|  | OsGSTU40 | Os12t0123200 | OsGSTF1 | Os01t0371200 |
|  | OsGSTF2 | Os01t0764000 | OsGSTF3 | Os03t0135300 |
|  | OsGSTF4 | Os01t0933900 | OsGSTF5 | Os01t0369700 |
|  | OsGSTF6 | Os01t0369700 | OsGSTF7 | Os01t0370200 |
|  | OsGSTF8 | Os01t0764000 | OsGSTF9 | Os01t0371400 |
|  | OsGSTF10 | Os01t0371500 | OsGSTF11 | Os01t0353400 |
|  | OsGSTF12 | Os01t0374000 | OsGSTF13 | Os01t0372400 |
|  | OsGSTF14 | Os03t0134900 | OsGSTF15 | Os03t0135100 |
|  | OsGSTF16 | Os05t0148900 | OsGSTZ1 | Os12t0210200 |
|  | OsGSTZ2 | Os12t0210300 | OsGSTZ3 | Os02t0564000 |
|  | OsGSTZ4 | Os11t0245100 | OsGSTT1 | Os11t0588300 |
|  | OsDHAR1 | Os06t0232600 | OsTCHQD1 | Os04t0435500 |

**Table S5** List of gene sequences used to construct phylogenetic tree.

|  | Species | Gene ID | Species | Protein ID |
| --- | --- | --- | --- | --- |
| Dicotyledonous | Eggplant | XM_006365078.2 | Castor | XM_002518140.4 |
|  | Dianthus | X58390.1 | Poppy | XM_026555322.1 |
|  | Beet | XM_010690907.3 | Spinach | XM_021984377.1 |
|  | Kale | JN656712.1 | Chinese cabbage | JX110775.1 |
|  | Rape | AY208158.1 | Radish | XM_018617115.1 |
|  | Arabidopsis | AB039930.1 | Pumpkin | XM_023135801.1 |
|  | Cucumber | XM_011661050.2 | Momordica charantia | XM_022295865.1 |
|  | Gourd | XM_039042775.1 | Cotton | AF159229.1 |
|  | Hibiscus | XM_039199879.1 | Euphorbia | MW718854.1 |
|  | Tung oil | GR218201.1 | Rubber | JF729318.1 |
|  | Rose | XM_018902949.1 | Raspberry | GT029978.1 |
|  | Strawberry | XM_036646500.1 | Apple | JZ822393.1 |
|  | Pear | DQ901400.1 | Peach | XM_007200428.2 |
|  | Plum bossom | XM_016792471.1 | Plums | XM_007200428.2 |
|  | Alfalfa | AB040439.1 | Poplar | JF422812.1 |
|  | Salix | MK300944.1 | Chestnut | DQ673317.1 |
|  | Mulberry | XM_010102984.2 | Jujube | HM345954.3 |
|  | Grape | NM_001280940.1 | Soybean | NM_001251762.2 |
|  | Pea | AB087837.1 | Orange | NM_001288861.1 |
|  | Persimmon | AB435082.1 | Walnut | KT351091.1 |
|  | Carrot | XM_017396379.1 | Tobacco | D10524.1 |
|  | Capsicum | XM_016720655.2 | Sunflower | XM_022113275.2 |
|  | Cocoa | XM_007050788.2 | Eucalyptus | XM_010071655.3 |
|  | Olive  Tomato | XM_023025104.1  Solyc09g011630 | Chickpea | XM_012716571.2 |
| Monocotyledon | Oil palm | KF516927.1 | Rice | AJ486976.1 |
|  | Wheat | AF002211.1 | Barley | AB207242.1 |
|  | Sorghum | XM_021446718.1 | Sugar cane | JN591762.1 |
|  | Maize | NM_001156789.1 | Lilium regale | KM879340.1 |
|  | Pineapple | XM_020256604.1 | lettuce | XM_023888908.2 |
|  | Banana | KC261938.1 | Asparagus | XM_020393674.1 |
|  | Onion | AB300334.1 | Garlic | MH892343.1 |
|  | Ginger | XM_042594696.1 | Switchgrass | XM_039946577.1 |
|  | Reed | EU719194.1 | Green bristlegrass | XM_034721410.1 |
|  | Lemna | KM247621.1 | Phoenix dactylifera | XM_008782813.4 |
|  | Eragrostis | KM604231.1 | Millet | XM_004960717.4 |
|  | Tulips | KF134536.1 | Guinea yam | XM_039275788.1 |
|  | Hyacinth | AY389775.1 | Butterfly orchid | XM_020744136.1 |
|  | Aegilops | XP_020161671.1 | Panicum miliaceum | RLM86960.1 |
|  | Carex littledalei | KAF3329801.1 | Dendrobium catenatum | PKU87189.1 |
|  | Lolium perenne | AMY26594.1 |  |  |

**Table S6** List of primer sequences used for ChIP-qPCR analysis.

| Application | **primer** |
| --- | --- |
| *SlGSTU43*-ChIP-p2  Actin7 | **Forward**:CGTGACGGAAAGAAGTTGG  **Reverse**: GTTTCTTCTCAATTGAATATGCTAG  **Forward**: TGTCCCTATTTACGAGGGTTATGC  **Reverse**: CAGTTAAATCACGACCAGCAAGAT |

**Table S7** List of prey TFs.

| **Gene ID** | **Gene ID** |
| --- | --- |
| 1.Solyc09g090130 (SlMYB4)  2.Solyc05g007160 (SlMYB88)  3. Solyc08g081500  4. Solyc08g067360  5. Solyc01g096370  6. Solyc08g005050 | 7. Solyc03g078120  8. Solyc01g009860  9. Solyc01g079480  10. Solyc01g008880  11. Solyc06g050840  12. Solyc10g054330 |
